# MYO1F regulates antifungal immunity by regulating acetylation of microtubules

**DOI:** 10.1073/pnas.2100230118

**Published:** 2021-07-23

**Authors:** Wanwei Sun, Xiaojian Ma, Heping Wang, Yanyun Du, Jianwen Chen, Huijun Hu, Ru Gao, Ruirui He, Qianwen Peng, Zhihui Cui, Huazhi Zhang, Junhan Wang, Xinming Jia, Bradley N. Martin, Cun-Jin Zhang, Xiaoxia Li, Chenhui Wang

**Affiliations:** ^a^Key Laboratory of Molecular Biophysics of the Ministry of Education, National Engineering Research Center for Nanomedicine, College of Life Science and Technology, Huazhong University of Science and Technology, Wuhan 430074, China;; ^b^University-Affiliated Hospital, Huazhong University of Science and Technology, Wuhan 430074, China;; ^c^Shanghai Skin Disease Hospital, Tongji University School of Medicine, Shanghai 200443, China;; ^d^Department of Medicine, Brigham and Women’s Hospital, Harvard Medical School, Boston, MA 02115;; ^e^Department of Neurology of Nanjing Drum Tower Hospital Medical School, State Key Laboratory of Pharmaceutical Biotechnology, Nanjing University, Nanjing 210008, China;; ^f^Department of Inflammation and Immunity, Lerner Research Institute, Cleveland Clinic, Cleveland, OH 44106;; ^g^Bioinformatics Center, Wuhan Institute of Biotechnology, Wuhan 430070, China

**Keywords:** antifungal immunity, MYO1F, microtubules acylation, Sirt2 inhibitor

## Abstract

Opportunistic fungal infections have become one of the leading causes of death among immunocompromised patients, resulting in an estimated 1.5 million deaths each year worldwide. The molecular mechanisms that promote host defense against fungal infections remain elusive. Here, we find that Myosin IF (MYO1F), an unconventional myosin, promotes the expression of genes that are critical for antifungal innate immune signaling and proinflammatory responses. Mechanistically, MYO1F is required for dectin-induced α-tubulin acetylation, acting as an adaptor that recruits both the adaptor AP2A1 and α-tubulin *N*-*acetyltransferase* 1 to α-tubulin; in turn, these events control the membrane-to-cytoplasm trafficking of spleen tyrosine kinase and caspase recruitment domain-containing *protein 9*. Myo1f-deficient mice are more susceptible than their wild-type counterparts to the lethal sequelae of systemic infection with *Candida albicans*. Notably, administration of Sirt2 deacetylase inhibitors, namely AGK2, AK-1, or AK-7, significantly increases the dectin-induced expression of proinflammatory genes in mouse bone marrow–derived macrophages and microglia, thereby protecting mice from both systemic and central nervous system *C. albicans* infections. AGK2 also promotes proinflammatory gene expression in human peripheral blood mononuclear cells after Dectin stimulation. Taken together, our findings describe a key role for MYO1F in promoting antifungal immunity by regulating the acetylation of α-tubulin and microtubules, and our findings suggest that Sirt2 deacetylase inhibitors may be developed as potential drugs for the treatment of fungal infections.

## Significance

Each year, the global mortality rates for fungal diseases now exceed those for malaria and breast cancer and are currently comparable to those for tuberculosis and HIV. The limited scope of currently available antifungal drugs is the major factor underlying the observed high mortality rate. Here, we provide evidence that Myosin IF (MYO1F) plays a critical role in the mediating of signaling molecules “trafficking from membrane to cytoplasm,” and this process is essential for the antifungal signaling pathway activation. Moreover, we provide evidence that Sirt2 deacetylase inhibitors promote antifungal immunity and protect mice from lethal *Candida albicans* infection, which indicates that the Sirt2 could be a good therapeutic target for the antifungal drug development.

Each year, invasive fungal infections lead to the deaths of ∼1.5 million people worldwide; the effects of fungal infections are particularly severe among patients with compromised immune systems, such as patients infected with HIV, patients receiving immunosuppressive therapy for rheumatological diseases, and patients receiving chemotherapy for both liquid and solid malignancies (1–3). *Candida albicans* is the most frequently isolated fungal pathogen in all parts of the world, and it has become one of the leading causes of infection in hospital settings (1). The limited scope of the currently available antifungal drugs and widespread drug resistance are both major factors that lead to the observed high mortality rate. The global mortality rate for fungal diseases now exceeds those for malaria and breast cancer and is currently comparable to those for tuberculosis and HIV (2, 4, 5). The central nervous system (CNS) is also commonly invaded by fungal species, and fungal infections of the CNS have high mortality rates of over 50% (6, 7). It was reported that patients with CARD9 deficiency exhibit impaired neutrophil accumulation and uncontrolled CNS *Candida* infection (8). The treatment of fungal infections in the CNS is associated with great challenges due to impaired drug penetration and resistance to antifungal treatments. As such, a deeper understanding of host antifungal immunity is crucial for the development of novel drugs and treatment options.

C-type lectin receptors (CLRs) play a critical role in the detection and recognition of fungal species, such as *Candida*, during infection (9, 10). CLRs include dectin-1, dectin-2, dectin-3, Mincle, and mannose receptor C type 1 (MRC1). The recognition of various components of the fungal cell wall, including β-glucan, α-mannan, hyphal mannose, and glycolipids, by these receptors initiates antifungal immune responses (11–15). In addition to CLRs, Toll-like receptor 2 (TLR2) is also involved in the recognition of fungal pathogens and the activation of protective immunity. The recognition of fungal cell wall components by various CLRs results in the activation of spleen tyrosine kinase (Syk) in macrophages and dendritic cells (DCs), thereby triggering the activation of the CARD9–BCL10–MALT1 (CBM) complex-dependent nuclear factor-κB (NF-κB) and mitogen-activated protein kinase (MAPK) signaling pathways, which is followed by the synthesis and release of proinflammatory cytokines and chemokines. These include interleukin-6 (IL-6), IL-1β, IL-23A, IL-12A, CXCL1, and granulocyte-macrophage colony-stimulating factor (GM-CSF), among others. Many of the new molecules involved in antifungal signaling pathways, such as SHP-2, PLC-γ2, and kinase PKC-δ, have been recently identified (16–18). Lysosome-mediated degradation was also reported to be a potential immune defense strategy that promotes the clearance of invasive fungi (19–21). Interestingly, and in parallel with studies carried out in mice, loss-of-function mutations in the *DECTIN-1*, *CARD9*, *STAT3*, *RORC*, *IL-17A*, *IL-17F*, *IL-17RA*, *IL-17RC*, *TRAF3IP2*, and *JNK1* genes have been identified in patients with chronic mucocutaneous candidiasis (CMC), which indicates that both innate immunity and adaptive immunity are involved in the human antifungal immune response (22–29).

MYO1F, an unconventional myosin, is predominantly expressed in cells of the mammalian immune system. In recent findings, MYO1F was identified as critical for immune cell motility and innate host defense against infection with *Listeria monocytogenes* (30). MYO1F and MYO1E play redundant roles in promoting macrophage-mediated phagocytosis by controlling actin dynamics (30–34). Mutations in the coding sequence of the *MYO1F* gene or *MYO1F* gene fusions have been shown to be associated with familial non-medullary thyroid cancer and peripheral T cell lymphomas, suggesting that MYO1F may also be involved in human tumorigenesis (35–38). Here, we report that MYO1F plays a critical role in dectin-activated signaling by regulating α-tubulin acetylation, which in turn directs the translocation of Syk and CARD9 from the cell membrane to the cytoplasm for the recruitment of downstream signaling molecules, such as IKK-α/β. Among our findings, we report that Myo1f-deficient mice are susceptible to lethal systemic infection with *Candida albicans* and that hematopoietic cell–expressed Myo1f plays a major role in antifungal immunity. Notably, we find that administration of inhibitors of the deacetylase Sirt2, namely AGK2 or AK-1, promotes increases in dectin-activated signaling and proinflammatory gene expression. In vivo, these agents protect mice from the lethal sequelae of systemic *C. albicans* infection. Interestingly, a CNS-permeable Sirt2 inhibitor, AK-7, shows a therapeutic effect on CNS *C. albicans* infection. Overall, these results indicate that MYO1F plays a critical role in innate antifungal immunity by regulating Syk/CARD9 “membrane to cytoplasm trafficking,” that the deacetylase Sirt2 is a good therapeutic target for antifungal drug development, and that inhibitors of Sirt2 may be developed as potential drugs for the treatment of fungal infection.

## Results

### MYO1F Is Required for the Activation of Antifungal Signaling in Human THP-1 Cells.

**Fig. 1.**
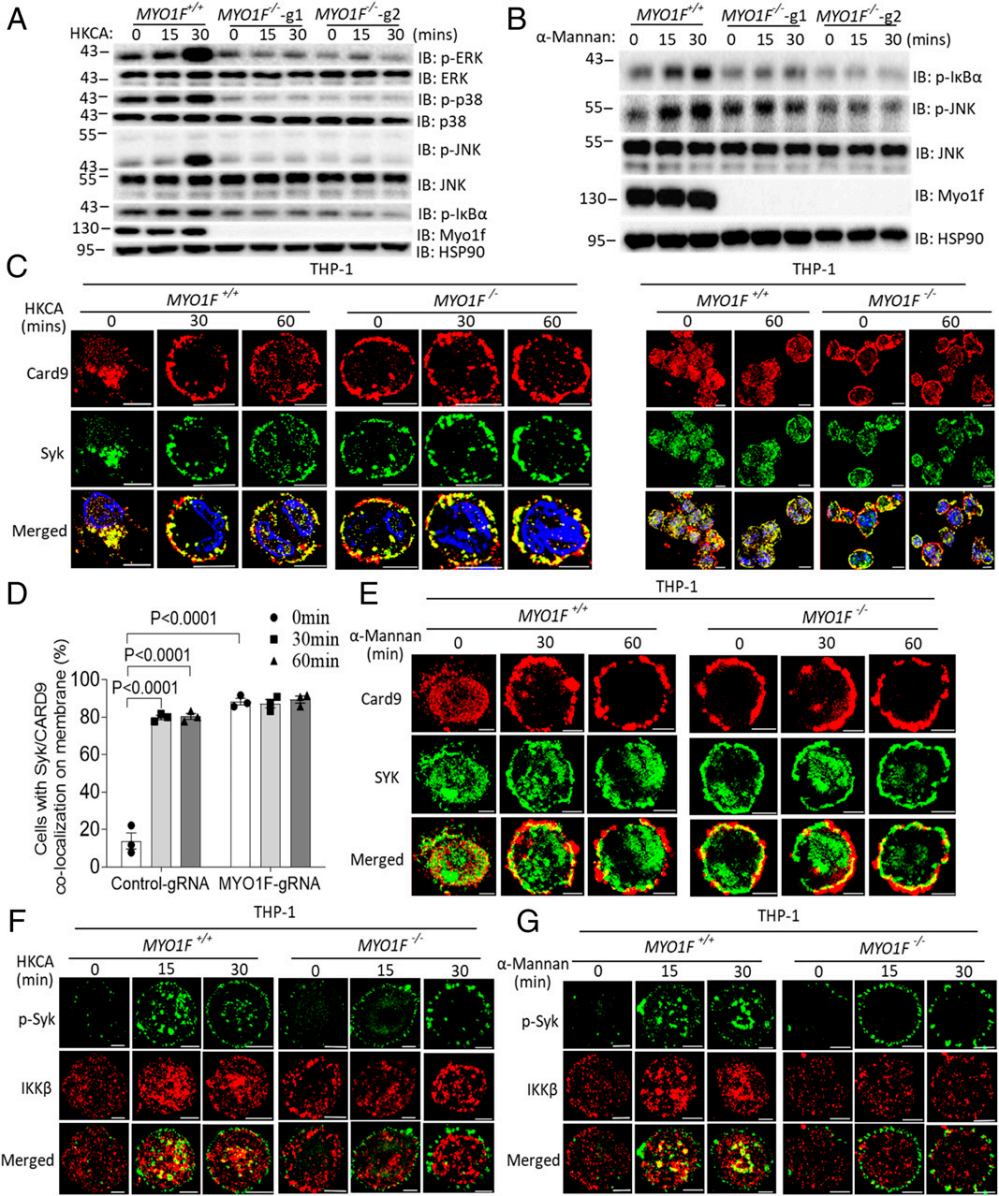
MYO1F is required for antifungal signaling activation in human THP-1 cells. (*A* and *B*) Control THP-1 cells or MYO1F-KO THP-1 cells were left untreated or stimulated with HKCA (MOI = 2) (*A*) or α-mannan (100 μg/mL) (*B*) for the indicated times followed by Western blot analysis of indicated proteins. (*C* and *D*) Control THP-1 cells or MYO1F-KO THP-1 cells were stimulated as in *A* for the indicated times followed by immunofluorescence staining of indicated proteins. The data in *Left* and *Right* was presented by using different magnifications of microscope analysis for the same batch of experiment. (Scale bar: 5 μm.) The cells with Syk/CARD9 colocalization were calculated as percentages, which was described in *Materials and Methods*. (*E*–*G*) Control THP-1 cells or MYO1F-KO THP-1 cells were stimulated with α-mannan (100 μg/mL, *E*), HKCA (*F*, MOI = 2), or Mannan (*G*, 100 μg/mL) for the indicated times followed by immunofluorescence staining of indicated proteins. (Scale bar: 5 μm.) One-way ANOVA for *D*. Data are representative of three independent experiments.

In our previous study, we found that the activation of Rho GTPase-activating protein (TAGAP) in T cells is a critical component of the host-mediated antifungal immune signaling pathway (39). As part of an ongoing effort to understand the mechanistic role of TAGAP in antifungal signaling pathways, we performed glutathione-S-transferase (GST) pulldown assay and mass spectrometry to identify TAGAP-interacting proteins. Interestingly, Myosin 1f (MYO1F), a member of the large family of unconventional myosin proteins, was identified by mass spectrometry as a TAGAP-interacting protein (Datasets S1–S3). Next, to explore the functional role of MYO1F in antifungal signaling, we generated MYO1F-deficient THP-1 cells using the CRISPR-Cas9 method. We found that the induction of NF-κB and MAPK signaling by heat-killed *C. albicans* was completely eliminated in the absence of MYO1F (Fig. 1*A*). Heat-killed *C. albicans* expresses β-glucans, which are dectin-1 ligands, on its cell wall (40). Therefore, we examined whether MYO1F might also be involved in Dectin-2/Dectin-3 signaling by stimulating cells with α-mannan, the Dectin-2/3–specific ligand (15). We found that α-mannan–induced NF-κB and MAPK signaling was also abolished in MYO1F-deficient THP-1 cells compared to wild-type (WT) control THP-1 cells (Fig. 1*B*). The live *C. albicans* cell wall contains β-glucans and chitin, which are oriented toward the inside of the cell wall, and the outer layer is enriched with α-mannan (41). For a more realistic simulation of fungal infection, we stimulated THP-1 cells with live *C. albicans* and examined whether MYO1F deficiency had any impact on signaling activation and proinflammatory gene expression. We found that live *C. albicans*–induced NF-κB and MAPK signaling was also significantly defective in MYO1F-deficient THP-1 cells compared to WT cells (*SI Appendix*, Fig. S1*A*). Taken together, these data suggest that MYO1F plays a critical role in the activation of antifungal signaling pathways.

Previous studies have reported Syk translocation from the cytoplasm to the plasma membrane following activation by dectin (16). Consistent with previous reports, we observed Syk and CARD9 translocation from the cytoplasm to the plasma membrane in WT THP-1 cells in response to both heat-killed *C. albicans* and α-mannan. Interestingly, Syk and CARD9 were detected at the membrane of MYO1F-deficient THP-1 cells regardless of whether the cells were stimulated with dectin ligand (Fig. 1 *C*–*E*). These data suggest that the membrane-to-cytoplasm trafficking of Syk and CARD9 may be important for antifungal signaling, and the specific defect in MYO1F-deficient THP-1 cells may be related to the retention of these signaling molecules at the membrane. We did not observe the phenomenon of Syk and CARD9 membrane retention in TAGAP-deficient cells, which indicates that MYO1F-mediated antifungal signaling is independent of TAGAP (39). It was reported that activated Syk recruits the CARD9–BCL10–MALT1 (CBM) complex to activate the IKK-α/β complex for nuclear factor-κB (NF-κB) signaling pathway activation (16, 42–44). Next, we found that phosphorylated Syk localized to the plasma membrane and colocalized with IKK-β in the cytoplasm of WT THP-1 cells after stimulation with heat-killed *C. albicans* or α-mannan, while in MYO1F-knockout (KO) THP-1 cells, phosphorylated Syk was retained at the plasma membrane and did not show any colocalization with IKK-β (Fig. 1 *F* and *G*). Overall, these data indicate that MYO1F plays a critical role in antifungal signaling pathways by regulating the “membrane to cytoplasm” trafficking of both Syk and CARD9.

### Myo1f Is Required for Antifungal Signaling Activation in Mouse Macrophages.

**Fig. 2.**
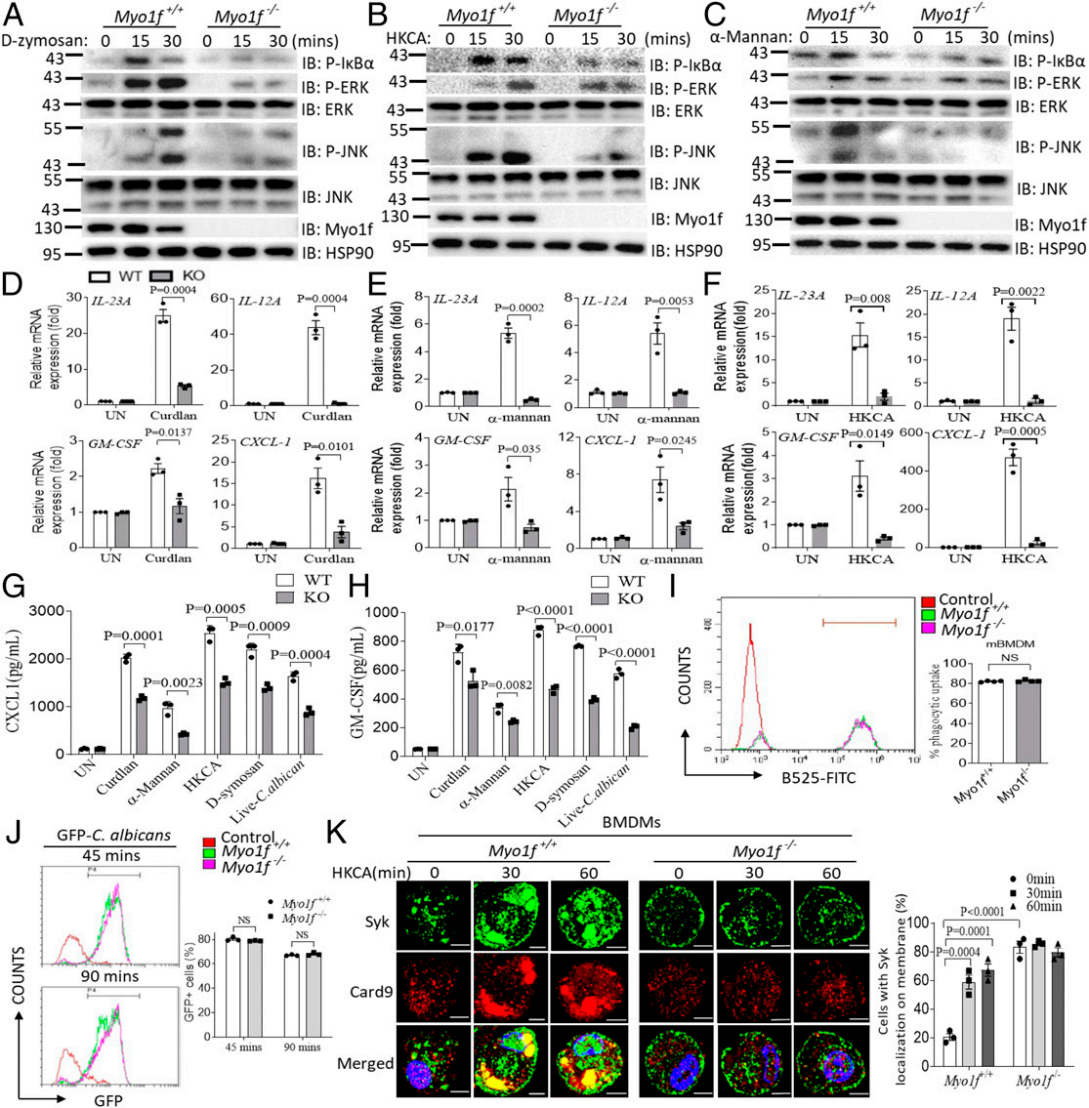
Myo1f is required for antifungal signaling activation in mouse BMDMs. (*A*–*C*) BMDMs from WT control mice or Myo1f-deficient mice were stimulated with D-zymosan (100 μg/mL) (*A*), HKCA (MOI = 2) (*B*), or α-mannan (100 μg/mL) (*C*) for the indicated time followed by Western blot analysis of indicated proteins expression. (*D*–*F*) BMDMs from WT control mice or Myo1f-deficient mice were stimulated with curdlan (100 μg/mL) (*D*), α-mannan (100 μg/mL) (*E*), and HKCA (MOI = 2) (*F*) for the indicated times followed by RT and real-time PCR analysis of indicated gene expression. (*G* and *H*) BMDMs from WT control mice or Myo1f-deficient mice were stimulated with curdlan (100 μg/mL), α-mannan (100 μg/mL), HKCA (MOI = 2), D-zymosan (100 μg/mL), or live *C. albicans* (MOI = 1) for 24 h followed by enzyme-linked immunosorbent assay (ELISA) analysis of CXCL1 (*G*) or GM-CSF (*H*) production. (*I* and *J*) Phagocytosis of WT mice or Myo1f-KO BMDMs was evaluated by the method described in *Materials and Methods*. *n* = 4 for *I* and *n* = 3 for *J*. (*K*) BMDMs from WT control mice or Myo1f-deficient mice were left untreated or stimulated with HKCA (MOI = 2) for the indicated time followed by immunofluorescence staining of indicated proteins. (Scale bar: 5 μm.) Cells with Syk membrane localization were calculated as percentages, which was described in *Materials and Methods* (*Right*). Two-tailed unpaired Student’s *t* test (*D*–*J*) and one-way ANOVA for *K*. Error bars represent SEM of biological replicates for *D*–*H*. Data are representative of three independent experiments.

We next explored whether the functional role of Myo1f was conserved in mouse macrophages. Myo1f-deficient mice were generated using CRISPR-Cas9 methods (*SI Appendix*, Fig. S1*B*). We then examined the role of Myo1f in antifungal signaling in mouse bone marrow–derived macrophages (BMDMs). The findings from mouse BMDMs were consistent with those from human THP-1 cells. Specifically, the signaling induced by the dectin-1 ligand D-zymosan and heat-killed *C. albicans* was dramatically reduced in Myo1f-deficient BMDMs compared to BMDMs from WT mice, and signal activation induced by the dectin-2/dectin-3 ligand α-mannan was also substantially impaired (Fig. 2 *A*–*C*). The proinflammatory cytokine expression induced by curdlan, α-mannan, or heat-killed *C. albicans* (HKCA) was also significantly reduced in Myo1f-deficient BMDMs compared to control cells (Fig. 2 *D*–*F*). Consistently, CXCL1 and GM-CSF cytokine production was significantly reduced in the Myo1f-deficient BMDMs compared to WT BMDMs after treatment with curdlan, α-mannan, HKCA, D-zymosan, and live *C. albicans* (Fig. 2 *G* and *H*). Myo1f-deficient BMDMs had significant defects compared to WT cells in terms of signaling activation and proinflammatory cytokine expression after live *C. albicans* stimulation (*SI Appendix*, Fig. S1 *C* and *D*). Together, these data indicate that Myo1f also plays a critical role in antifungal innate immunity in mouse macrophages.

Myo1f and Myo1e play an important role in phagocytosis (31). To exclude the possibility that the signaling defect in Myo1f-KO macrophages is due to defects in phagocytosis, we examined whether Myo1f-KO cells have any defect in phagocytosis. We did not detect any difference in phagocytic uptake between WT and Myo1f-KO BMDMs by two different methods, which is consistent with a previous report that Myo1f deficiency alone does not affect phagocytosis (31) (Fig. 2 *I* and *J*). The antifungal immune response can induce reactive oxygen species (ROS) production to distinguish bacterial and fungal propagation (45). Defects in ROS production lead to chronic granulomatous disease, a disorder characterized by recurrent bacterial and fungal infections (46). Next, we explored whether MYO1F deficiency had any impact on ROS production. Consistent with defective signaling activation, we found that ROS production was significantly impaired in Myo1f-KO THP-1 cells compared to WT cells after treatment with HKCA or α-mannan (*SI Appendix*, Fig. S1*E*). Consistently, in vitro fungal killing was significantly attenuated in MYO1F-KO THP-1 cells compared to WT control cells (*SI Appendix*, Fig. S1*F*). We did not observe significant defects in Myo1f-KO macrophages compared to control macrophages after stimulation with the TLR4 and TLR-2 ligands lipopolysaccharide (LPS) and lipoteichoic acid (LTA), which suggests that MYO1F specifically regulates antifungal immunity (*SI Appendix*, Fig. S1 *G* and *H*).

Consistent with our findings in human THP-1 cells, we detected Syk translocation to the plasma membrane after stimulation with HKCA. In Myo1f-deficient BMDMs, Syk was retained at the plasma membrane regardless of whether the cells were stimulated with HKCA (Fig. 2*K*). In contrast, no translocation of CARD9 was detected in WT control BMDMs in response to stimulation; instead, CARD9 colocalized with Syk and formed large aggregates within the cytoplasm in response to HKCA. There was also no obvious colocalization of CARD9 and Syk in Myo1f-deficient BMDMs following stimulation with HKCA (Fig. 2*K*). Together, these data indicate that in mouse BMDMs, Myo1f plays an essential role in antifungal signaling by regulating the “membrane to cytoplasm trafficking” of Syk.

### MYO1F Interacts with α-tubulin.

**Fig. 3.**
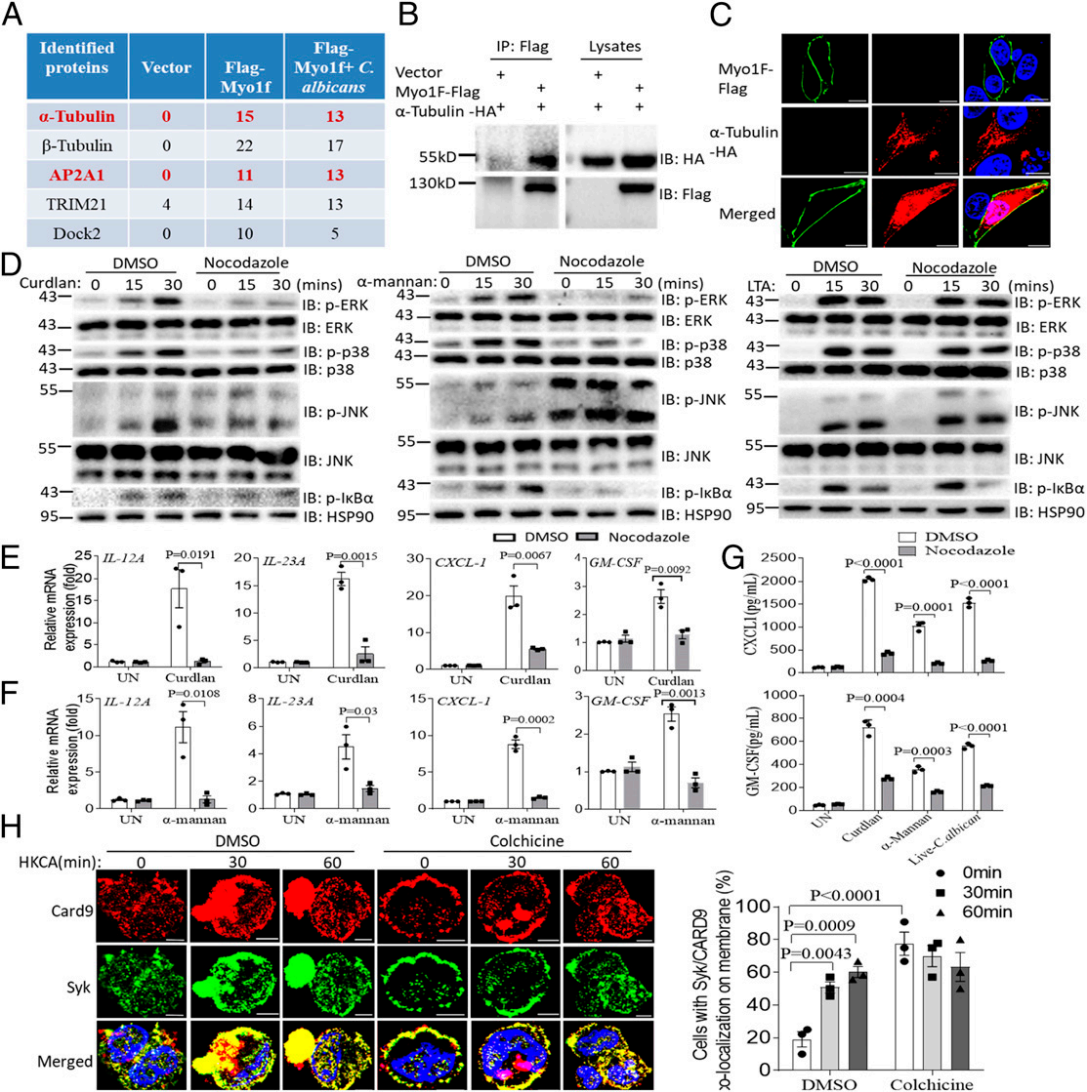
MYO1F interacts with α-Tubulin and AP2A1. (*A*) MYO1F-interacting proteins identified by mass spectrometry are shown. (*B*) HEK293T cells were transfected with indicated plasmids, and cell lysates were immunoprecipitated with anti-Flag antibody followed by immunoblot analysis for indicated proteins. (*C*) HeLa cells were transfected with indicated plasmids followed by immunofluorescence staining of anti-Flag or anti-HA antibodies. (*D*) BMDMs from WT mice were pretreated with nocodazole (10 μM) for 1 h followed by stimulation with curdlan (100 μg/mL), α-mannan (100 μg/mL), or LTA (100 μg/mL) for the indicated times. Cell lysates were analyzed by Western blot for indicated proteins. (*E* and *F*) BMDMs from WT mice were pretreated with nocodazole (10 μM) for 1 h and then stimulated with curdlan (100 μg/mL, *E*) or α-mannan (100 μg/mL, *F*) for the indicated times and followed by real-time PCR analysis of indicated gene expression. (*G*) BMDMs from WT mice were pretreated with nocodazole (10 μM) for 1 h and then stimulated with curdlan (100 μg/mL) and α-mannan (100 μg/mL) for 24 h and followed by ELISA analysis of CXCL1 or GM-CSF production. (*H*) THP-1 cells were pretreated with colchicine (10 μM) for 1 h followed by stimulation with HKCA (MOI = 2) for the indicated times. Cells were analyzed by immunofluorescence staining for the indicated proteins. Cells with Syk/CARD9 membrane localization were calculated as percentages, which was described in *Materials and Methods* (*Right*). (Scale bar: 5 μm for *C* and *H.*) Two-tailed unpaired Student’s *t* test (*E*–*G*) and one-way ANOVA for *H*. Error bars represent SEM of biological replicates for *E*–*G*. Data are representative of three independent experiments.

In an effort to understand the specific mechanism(s) underlying the role of MYO1F in antifungal signaling, we performed immunoprecipitation followed by mass spectrometry to identify MYO1F-interacting proteins. Using this method, we identified both α- and β-tubulin as MYO1F-interacting proteins (Datasets S4–S6). The interaction between MYO1F and α-tubulin was confirmed by coimmunoprecipitation and colocalization studies (Fig. 3 *A*–*C*). Tubulin forms microtubules, which are a main component of the cytoskeleton, play important roles in many biological processes, and are critical for structural support, intracellular transport, and mitosis (47, 48). Microtubules provide platforms for the intracellular transport of various cellular cargos, including organelles (48). Microtubules have already been implicated in signal transduction related to the activation of NLRP3 inflammasomes (47). As such, we hypothesized that MY1OF may regulate the membrane translocation of Syk and CARD9 via α-tubulin–mediated microtubule assembly. Consistent with our hypothesis, we found that both curdlan- and α-mannan–induced phosphorylation of IκBα, ERK, and p38 was dramatically reduced in response to treatment with the microtubule assembly inhibitors nocodazole and colchicine, while TLR2 ligand-induced signaling was unaffected (Fig. 3*D* and *SI Appendix*, Fig. S2). Interestingly, curdlan- and α-mannan–induced phosphorylation of JNK was increased in response to colchicine or nocodazole, which is consistent with previous reports (49, 50) (Fig. 3*D* and *SI Appendix*, Fig. S2). Curdlan- and α-mannan–induced proinflammatory cytokine gene expression was also significantly impaired after treatment with nocodazole (Fig. 3 *E* and *F*). Consistently, CXCL1 and GM-CSF cytokine production was also significantly reduced in the nocodazole-treated BMDMs compared to control-treated BMDMs after stimulation with curdlan, α-mannan, or live *C. albicans* (Fig. 3*G*). Notably, Syk and CARD9 were retained at the plasma membrane of unstimulated cells, similar to what was found in MYO1F-deficient THP-1 cells (Fig. 3*H*). Together, these data suggest that MYO1F regulates antifungal signaling via a direct impact on α-tubulin–mediated microtubule assembly.

### Dynein Mediates “Membrane to Cytoplasm Trafficking” in Response to Fungal Stimulation.

**Fig. 4.**
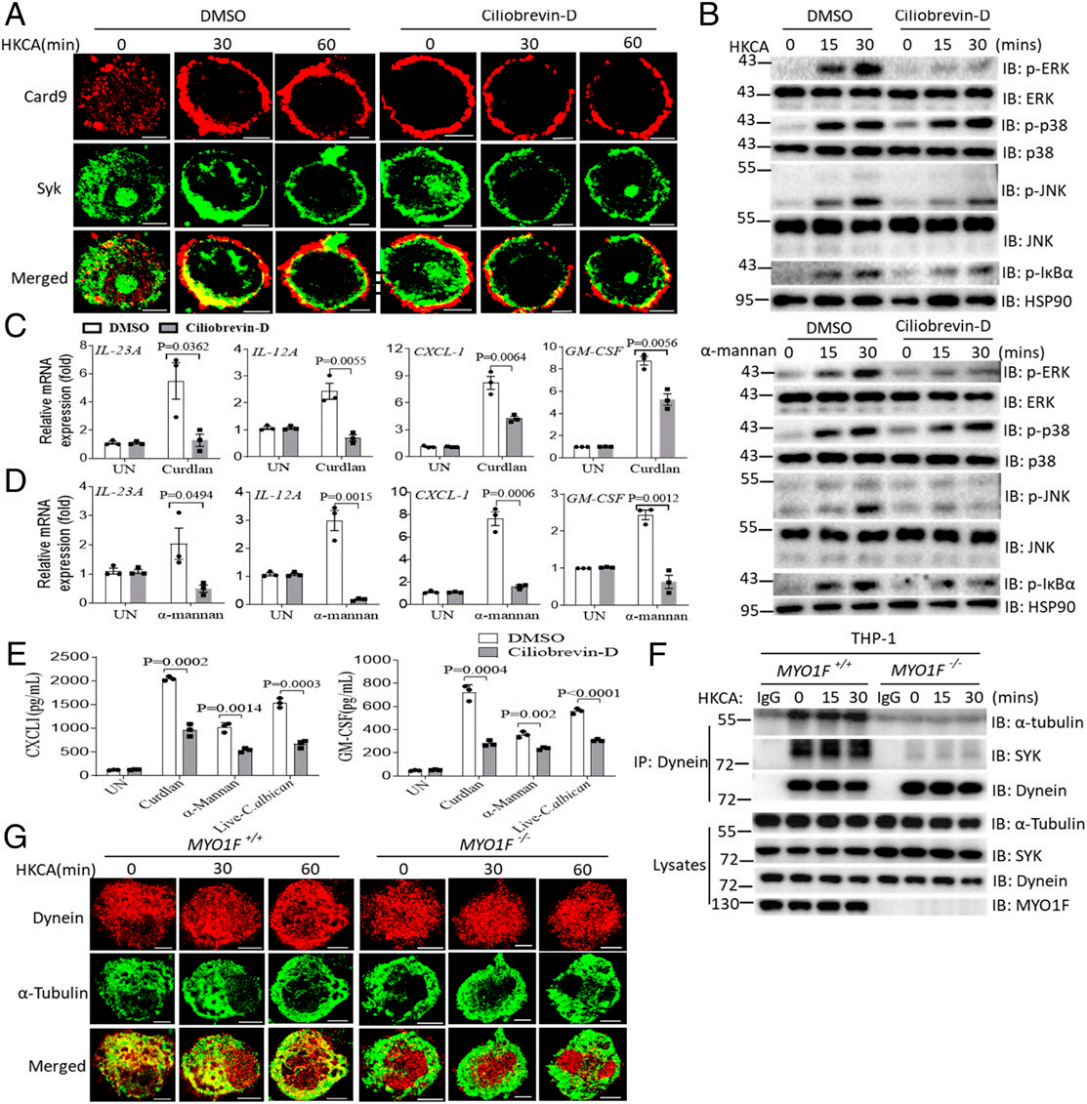
Dynein plays a critical role for the membrane trafficking of Syk and CARD9. (*A*) THP-1 cells were pretreated with ciliobrevin-D (10 μM) for 1 h followed by stimulation of HKCA (MOI = 2) for the indicated times. Cells were analyzed by immunofluorescence staining for indicated proteins. (Scale bar: 5 μm.) (*B*) BMDMs from WT mice were pretreated with ciliobrevin-D (10 μM) for 1 h followed by the stimulation of curdlan (100 μg/mL) or α-mannan (100 μg/mL) for the indicated times. Cell lysates were analyzed by Western blot for indicated proteins. (*C* and *D*) BMDMs from WT mice were pretreated with ciliobrevin-D (10 μM) for 1 h followed by stimulation of curdlan (100 μg/mL) (*C*) or α-mannan (100 μg/mL) (*D*) for the indicated times, and proinflammatory cytokine expression was analyzed by RT and real-time PCR. (*E*) BMDMs from WT mice were pretreated with ciliobrevin-D (10 μM) for 1 h followed by stimulation of curdlan (100 μg/mL), α-mannan (100 μg/mL), or live *C. albicans* (MOI = 1) for the 24 h and followed by ELISA analysis of CXCL1 or GM-CSF production. (*F*) WT control THP-1 cells or MYO1F-KO THP-1 cells were stimulated with HKCA (MOI = 2) for the indicated times, and cell lysates were immunoprecipitated by anti-Dynein antibody followed by Western blot analysis of indicated proteins. (*G*) WT control THP-1 cells or MYO1F-KO THP-1 cells were stimulated as in *A* for the indicated times followed by immunofluorescence staining of indicated proteins. (Scale bar: 5 μm.) Two-tailed unpaired Student’s *t* test (*C*–*E*). Error bars represent SEM of biological replicates for *C*–*E*. Data are representative of three independent experiments.

It was reported that signaling molecules are transported from the membrane to the cytoplasm via motor proteins, such as kinesin and dynein, along microtubules. Kinesin and dynein move in different directions in the cells; kinesin slides along the microtubule toward its “plus end,” while dynein slides along microtubules toward its “minus end” (51, 52). The finding that Syk and CARD9 were retained at the plasma membrane in Myo1f-deficient BMDMs suggests that dynein may play a role in this process. To explore this possibility, we used the dynein inhibitor ciliobrevin-D to block the translocation of signaling molecules from the plasma membrane to the cytoplasm and examined its impact on antifungal signaling activation (53). After the pretreatment of THP-1 cells with ciliobrevin-D, both Syk and CARD9 were retained at the plasma membrane, and fungal ligand-mediated activation had no impact on their subcellular localization. These findings were largely consistent with those observed in MYO1F-KO cells (Fig. 4*A*). Importantly, curdlan and α-mannan-induced signaling was markedly impaired following ciliobrevin-D pretreatment (Fig. 4*B*). Curdlan- and α-mannan–induced proinflammatory cytokine gene expression was also dramatically reduced in response to ciliobrevin-D treatment to an extent similar to that observed in MYO1F-KO cells (Fig. 4 *C* and *D*). CXCL1 and GM-CSF production was also significantly reduced in ciliobrevin-D–pretreated BMDMs compared to control-pretreated BMDMs after stimulation with curdlan, α-mannan, or live *C. albicans* (Fig. 4*E*). Published studies have shown that dynein can bind to microtubules in the presence of acetylated or tyrosinated α-tubulin (47, 54, 55). Immunoprecipitation and colocalization assays showed that the interaction between dynein and α-tubulin was almost abolished in MYO1F-KO THP-1 cells compared to control THP-1 cells after stimulation with HKCA, which indicates that the binding between dynein and α-tubulin is dependent on MYO1F (Fig. 4 *F* and *G*). These data also suggest that MYO1F may regulate the posttranslational modification of α-tubulin and microtubules for the binding of dynein.

### MYO1F Recruits the Adaptor Protein AP2A1 to Promote Fungi-Induced Acetylation of α-tubulin.

**Fig. 5.**
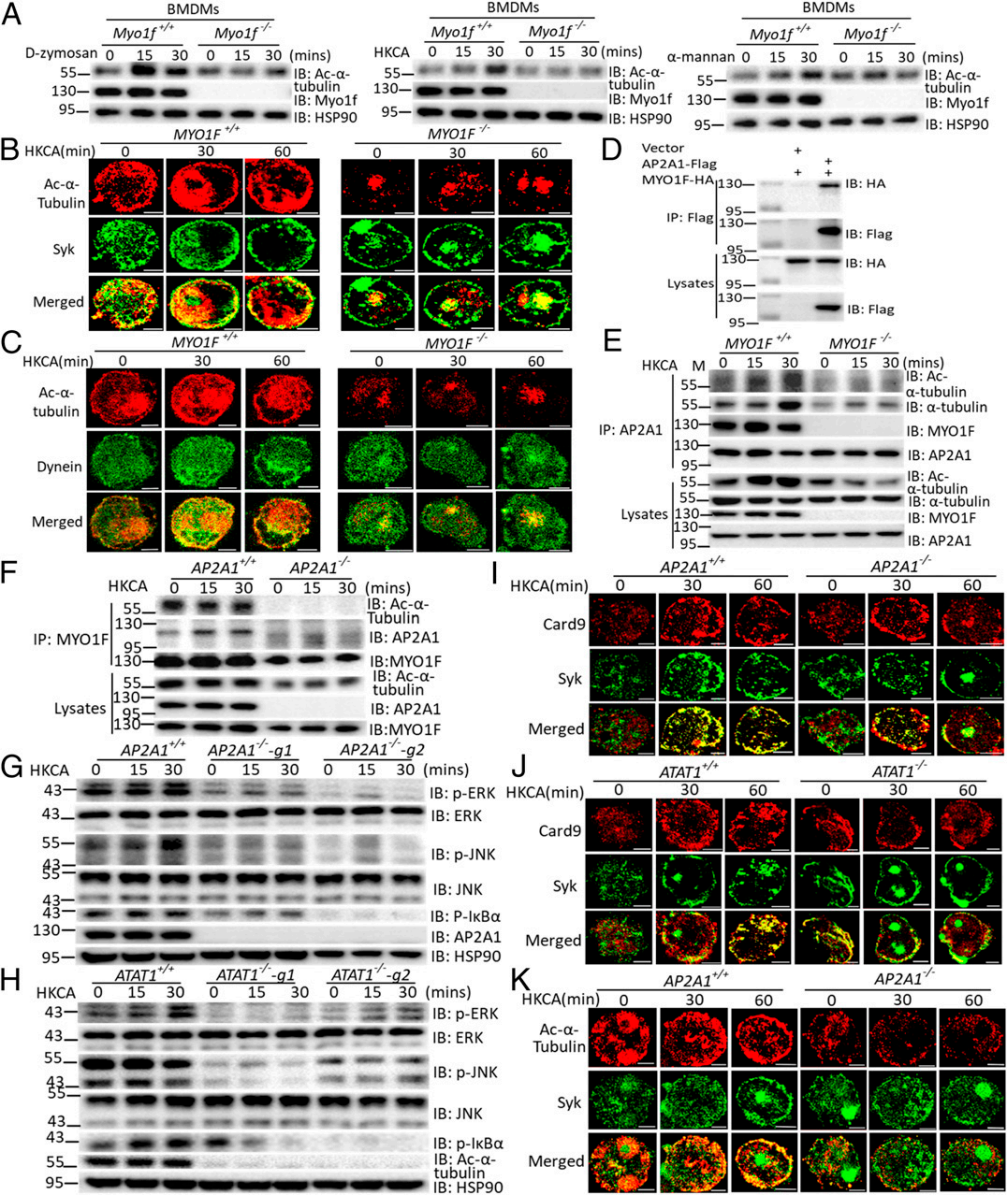
MYO1F is required for dectin-induced α-Tubulin acylation. (*A*) BMDMs from WT control mice or Myo1f-deficient mice were stimulated with D-zymosan (100 μg/mL), HKCA (MOI = 2), or α-mannan (100 μg/mL) for indicated times followed by Western blot analysis of indicated proteins. (*B* and *C*) Control THP-1 cells or MYO1F-KO THP-1 cells were stimulated with HKCA (MOI = 2) for the indicated times followed by immunofluorescence staining of indicated proteins. (Scale bar: 5 μm.) (*D*) HEK293T cells were transfected with indicated plasmids, and cell lysates were immunoprecipitated with anti-Flag antibody followed by immunoblot analysis for indicated proteins. (*E* and *F*) WT control THP-1 cells or MYO1F-KO THP-1 cells (*E*) and AP2A1-KO THP-1 cells (*F*) were stimulated with HKCA (MOI = 2) for the indicated times, and cell lysates were immunoprecipitated by anti-AP2A1 antibody (*E*) or anti-MYO1F antibody (*F*) followed by Western blot analysis of indicated proteins. (*G* and *H*) THP-1 cells infected with control guide RNA (gRNA) or AP2A1-gRNA (*G*) and αTAT1-gRNA (*H*) were stimulated with HKCA (MOI = 2) for the indicated times followed by Western blot analysis of indicated proteins. (*I*–*K*) THP-1 cells infected with control gRNA or AP2A1-gRNA (*I*), αTAT1-gRNA (*J*), and AP2A1-gRNA (*K*) were stimulated with HKCA (MOI = 2) for the indicated times followed by immunofluorescence staining of indicated proteins. (Scale bar: 5 μm for *B*, *C*, *I*, *J*, and *K.*) Data are representative of three independent experiments.

Posttranslational modifications of α-tubulin, including acetylation, polyglutamylation, and detyrosination, have been shown to significantly impact the function of microtubules (47, 48, 56). For example, dynein proteins can bind to acetylated α-tubulin to promote intracellular transport via microtubules (54, 56). Acetylated α-tubulin is a fundamental element of the mechanisms underlying the transport of the adaptor molecule ASC from the mitochondria to NLRP3 at the endoplasmic reticulum, thereby critically mediating inflammasome activation (47). Indeed, we found that D-zymosan, HKCA, and α-mannan each induced α-tubulin acetylation in WT BMDMs, while no α-tubulin acetylation was detected in Myo1f-deficient BMDMs (Fig. 5*A*). We next examined the acetylation of α-tubulin and Syk in control and MYO1F-deficient THP-1 cells and found that HKCA induced the translocation of acetylated α-tubulin from the cytoplasm to the plasma membrane in control THP-1 cells and the strong colocalization of acetylated α-tubulin and Syk in the cytoplasm (Fig. 5*B*). The membrane translocation of acetylated α-tubulin was nearly abolished in MYO1F-deficient THP-1 cells, with no apparent colocalization of acetylated α-tubulin and Syk after stimulation with HKCA (Fig. 5*B*). Notably, MYO1F deficiency had no detectable impact on the level of centromeric acetylated α-tubulin, which indicates that MYO1F regulates only the acetylation of membrane-localized microtubules (Fig. 5*B*). Consistent with our early data and the previous reports (Fig. 4 *F* and *G*) (54, 55), we found that acetylated α-tubulin strongly colocalized with dynein after HKCA stimulation in WT THP-1 cells; however, the colocalization between acetylated α-tubulin and dynein was almost abolished in MYO1F-KO THP-1 cells, which was due to the greatly reduced amount of acetylated α-tubulin localized to the cellular membrane (Fig. 5*C*). Together, these data strongly suggest that MYO1F is required for fungal-induced α-tubulin acetylation and, as such, plays a critical role in the membrane trafficking of signaling molecules, such as Syk and CARD9. To further examine the functional role of MYO1F in the regulation of α-tubulin acetylation, we utilized a nocodazole washout assay, which revealed that after nocodazole pretreatment for 5 h, the acetylation of α-tubulin was greatly reduced, which is also consistent with a previous report (57). Following nocodazole “washout,” we observed a recovery of the level of acetylated α-tubulin in WT THP-1 cells. Additionally, the recovery of acetylated α-tubulin was significantly slower in MYO1F-KO cells than in control cells, which was consistent across four independent MYO1F-KO cell clones (*SI Appendix*, Fig. S3 *A* and *B*). These data further confirm that MYO1F plays a critical role in promoting the acetylation of α-tubulin.

A previous study found that acetylase αTAT1 localizes to clathrin-coated pits (CCPs) beneath the cellular membrane through a direct interaction with adaptor-related protein complex 2 subunit alpha (AP2A1), which is required for microtubule acetylation (57). Interestingly, AP2A1 was also identified as a MYO1F-interacting protein in our mass spectrometry analysis (Fig. 5*D*). We therefore hypothesized that MYO1F may recruit acetylase αTAT1 to catalyze α-tubulin acetylation at the plasma membrane through AP2A1. We first confirmed the interaction between MYO1F and AP2A1 by coimmunoprecipitation (Fig. 5*D*). Next, we identified a region of MYO1F (amino acid [aa] 31 to 677) that is necessary for the interaction between MYO1F and α-tubulin (*SI Appendix*, Fig. S4*A*). Interestingly, findings from our immunofluorescence study indicate that this region of MYO1F was also critical for the interaction between MYO1F and AP2A1 (*SI Appendix*, Fig. S4*B*). Since aa 31 to 677 of MYO1F form a motor domain, we further divided this domain into two subdomains: the N terminus (aa 31 to 350) and the C terminus (aa 351 to 677) of the motor domain. Further mapping of the region of MYO1F that is critical for binding to α-tubulin and AP2A1 revealed that the N terminus (31 to 355 aa) of the MYO1F motor domain mediated binding to AP2A1, while the C terminus (351 to 677 aa) of the MYO1F motor domain was required for interaction with α-tubulin (*SI Appendix*, Fig. S4 *C*–*E*). These data suggest that MYO1F functions as an adaptor that mediates the interaction between AP2A1 and α-tubulin through distinct domains. To further confirm the adaptor role of MYO1F in antifungal signaling, we performed an endogenous immunoprecipitation study in control and MYO1F-deficient THP-1 cells. After stimulation with HKCA, we detected MYO1F, AP2A1, α-tubulin, and acetylated α-tubulin in a complex in WT THP-1 cells, while the recruitment of α-tubulin and acetylated α-tubulin to AP2A1 was dramatically reduced in MYO1F-deficient THP-1 cells (Fig. 5*E*). Similarly, the recruitment of acetylated α-tubulin to MYO1F was abolished in AP2A1-deficient THP-1 cells (Fig. 5*F*). These data indicate that MYO1F functions as an adaptor by recruiting AP2A1 to promote acetylation of α-tubulin.

We hypothesized that MYO1F recruits αTAT1 to α-tubulin via interaction with AP2A1, which in turn is necessary for the membrane trafficking of Syk and CARD9. To confirm this hypothesis, we examined antifungal signaling in AP2A1- and αTAT1-deficient THP-1 cells. We found that HKCA-activated antifungal signaling was substantially diminished in both AP2A1-deficient and αTAT1-deficient THP-1 cells compared to WT control THP-1 cells (Fig. 5 *G* and *H*). Syk was also retained at the plasma membrane in resting AP2A1- and αTAT1-deficient THP-1 cells in a manner similar to that observed in MYO1F-deficient cells; however, interestingly, the retention of CARD9 at the plasma membrane was not as obvious in AP2A1-KO THP-1 cells, which may be due to the compensatory effect of other AP2 subunits involved in this process (Fig. 5 *I* and *J*). Acetylated α-tubulin colocalized with Syk at the plasma membrane of control THP-1 cells following stimulation with HKCA, while no colocalization of acetylated α-tubulin and Syk was observed in AP2A1-deficient cells (Fig. 5*K*). These data suggest that acetylated α-tubulin promotes Syk translocation from the plasma membrane to the cytoplasm.

### Deacetylase Sirt2 Inhibitors Have a Therapeutic Impact on Systemic Fungal Sepsis.

**Fig. 6.**
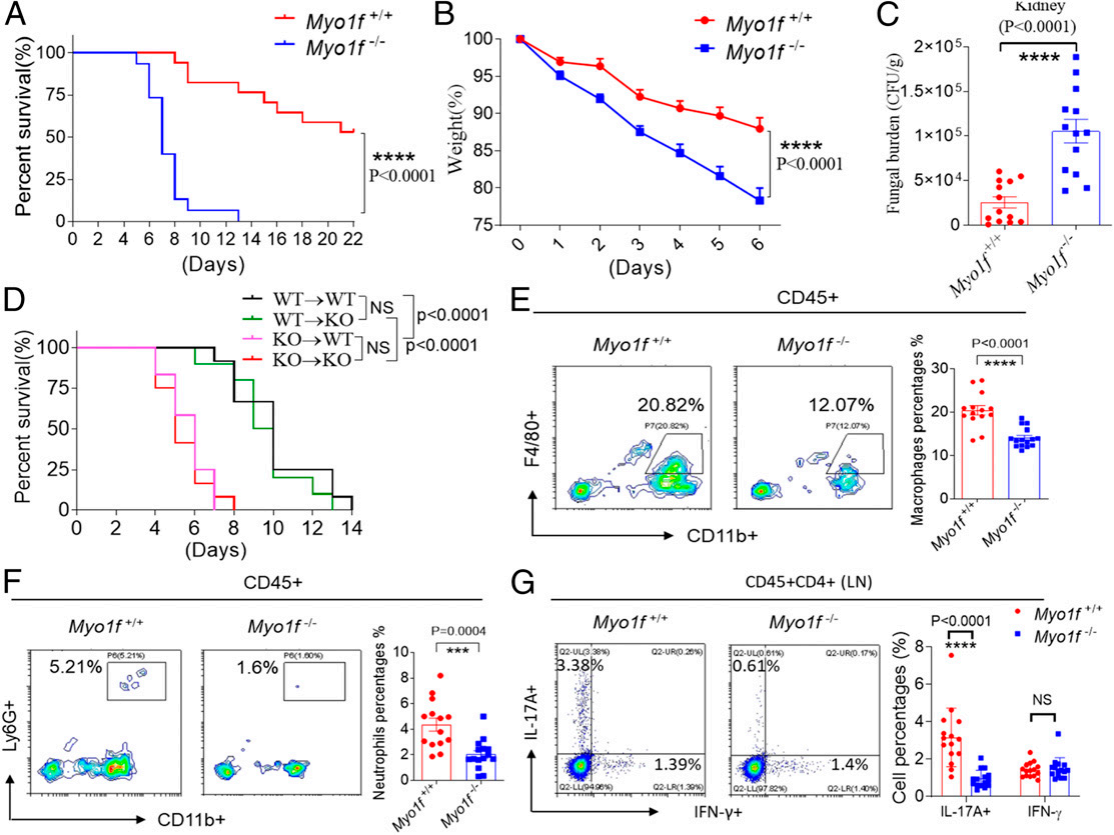
Myo1f KO mice were susceptible to fungal sepsis. (*A*–*C*) WT control mice or Myo1f-deficient mice were intravenously injected with live *C. albicans* (4 × 10^5^ cfu/100 μL 1× phosphate-buffered saline [PBS]), and mice survival curve (*A*), weight loss (*B*), and the fungal burden in the kidney (*C*) were shown. *n* = 15 (WT) and 17 (KO) for *A*; *n* = 16 (WT) and 14 (KO) for *B*; and *n* = 13 for *C*. (*D*) BM-chimeric mice were made by reconstituting lethally irradiated WT mice with syngeneic Myo1f-KO BM or Myo1f-KO mice with WT BM as described in *Materials and Methods*. Mice were intravenously injected with live *C. albicans* (3 × 10^5^ cfu/100 μL 1× PBS), and the mice survival curve was shown. *n* = 12 for each group. (*E* and *F*) WT mice or Myo1f KO mice were intravenously injected with live *C. albicans* (1 × 10^5^ cfu/100 μL 1× PBS), and mice were euthanized at day 1 after infection. Cells from kidneys were stained for flow cytometry analysis. *n* = 14 (WT) and 15 (KO) for *E* and *n* = 14 (WT) and 15 (KO) for *F*. (*G*) WT mice or Myo1f KO mice were intravenously injected with live *C. albicans* (1 × 10^5^ cfu/100 μL 1× PBS), and mice were euthanized at day 7 after infection. Cells from lymph nodes were stained as indicated for flow cytometry analysis. *n* = 15. ***P < 0.001; ****P < 0.0001 based on two-tailed unpaired Student’s *t* test (*C*, *E*, and *G*), two-way ANOVA (*B*), and log-rank (Mantel–Cox) test (*A* and *D*). NS: no significance. All error bars represent SEM of technical replicates. Data are pooled from three independent experiments.

Since Myo1f is ubiquitously expressed in mouse immune cells (http://biogps.org/#goto=genereport&id=17916), we first examined whether Myo1f-KO mice had any abnormality in immune cell abundance in the peripheral blood, lymph nodes, and spleen in the absence of infection. There were significantly decreased B cell numbers and increased neutrophil, monocyte, and macrophage numbers in the peripheral blood of Myo1f-KO mice compared to WT mice (*SI Appendix*, Fig. S5*A*). Consistently, there was also reduced B cell percentages in the spleen of Myo1f-KO mice compared to WT mice (*SI Appendix*, Fig. S5*B*). There were no significant changes in terms of Th17 or Th1 cell abundance in the lymph nodes or spleens between WT and Myo1f-KO mice in the absence of infection (*SI Appendix*, Fig. S5 *C* and *D*). These findings suggest that Myo1f may regulate mouse B cell development that might further play a role in the observed antifungal immunity defects in Myo1f KO mice as recently demonstrated by Itai Doron and colleagues that gut commensal fungi-induced antibody repertoire plays an indispensable role in antifungal immunity (58). Myo1f-KO mice were more susceptible to *C. albicans*–induced fungal sepsis than WT control mice, which was shown by the higher mortality rate, faster weight loss, and higher fungal burden of Myo1f-deficient mice compared to control mice (Fig. 6 *A*–*C*). Next, we explored whether myeloid cells or tissue cells expressing Myo1f play a major role in antifungal immunity by generating bone marrow (BM)-chimeric mice by reconstituting lethally irradiated WT mice with syngeneic Myo1f-KO BM or Myo1f-KO mice with WT BM. WT mice reconstituted with Myo1f-KO hematopoietic cells showed a phenotype similar to that of mice with total Myo1f deficiency in response to *C. albicans* infection (Fig. 6*D*, KO → WT versus KO → KO). These data indicate that hematopoietic cell-expressed Myo1f plays a major role in antifungal immunity. Macrophages and neutrophils play a critical role in antifungal innate immunity and function as the first line of defense to eliminate fungal infections (9, 59). After fungal infection, macrophages produce cytokines and chemokines, such as CXCL1 and GM-CSF, to recruit neutrophils and macrophages to eliminate infections (60, 61). Next, we explored whether there was any defect in local macrophage or neutrophil recruitment in Myo1f-KO mice after fungal infection. We found that there was significantly reduced macrophage and neutrophil infiltration in the kidneys of Myo1f-KO mice compared to WT control mice after systemic *C. albicans* infection (Fig. 6 *E* and *F*). There was a decreased Th17 cell abundance in the lymph nodes of Myo1f-KO mice compared to WT mice after fungal infection, while the abundance of Th1 cells was not different between WT mice and Myo1f-KO mice (Fig. 6*G*). Together, these data indicate that Myo1f plays an indispensable role in antifungal immunity.

**Fig. 7.**
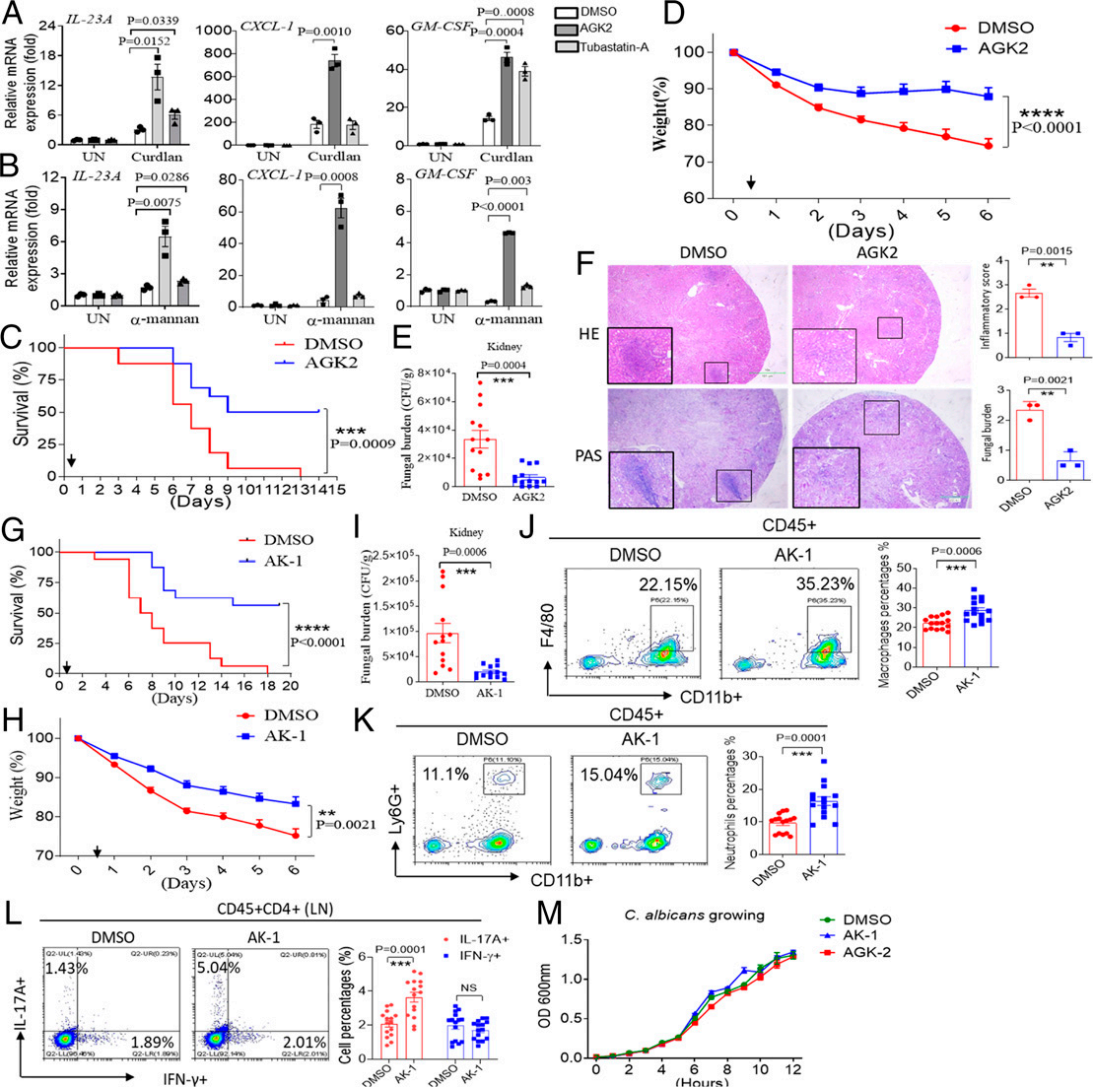
AGK2 and AK-1 have a therapeutic effect for mice systemic fungal sepsis. (*A* and *B*) BMDMs from WT mice were left untreated or pretreated with AGK2 (10 μM) or Tubastatin-A (10 μM) for one hour and stimulated with curdlan (100 μg/mL) (*A*) or α-mannan (100 μg/mL) (*B*) for indicated times followed by real-time PCR analysis of indicated gene expression. (*C*–*E*) WT mice were intravenously injected with live *C. albicans* (2 × 10^5^ cfu/100 μL 1× phosphate-buffered saline [PBS]) and intraperitoneally injected with DMSO or AGK2 (10 μg/50 μL) 12 h after *C. albicans* infection. Survival rate (*C*), weight loss rate (*D*), or fungal number in the kidney of mice were shown (*n* = 16 for *C*, *n* = 15 for *D*, and *n* = 13 for *E*). The arrow indicates the injection time of AGK2. (*F*) WT mice were treated as in *C*, and 48 h after *C. albicans* infection, mice were euthanized, kidneys were fixed, and sections were stained with hematoxylin and eosin (H&E) or periodic-acid Schiff (PAS). Renal inflammation and fungal burden were scored based upon H&E and PAS staining (*Right*), respectively. (*n* = 3). (*G*–*I*) WT mice were intravenously injected with live *C. albicans* (2 × 10^5^ cfu/100 μL 1× PBS) and intraperitoneally injected with DMSO or AK-1 (50 μg/100 μL 1× PBS) 12 h after *C. albicans* infection. Survival rate (*G*), weight loss rate (*H*), or fungal number in the kidney (*I*) of mice are shown [*n* = 16 for (*G*), *n* = 15 for (*H*), and *n* = 13 for (*I*)]. The arrow indicates the injection time of AK-1. (*J* and *K*) WT mice were treated as in *G*, and 2 d after *C. albicans* infection, mice were euthanized, and cells from kidneys were stained for flow cytometry analysis. *n* = 15. (*L*) WT mice were treated as in *G*, and 7 d after *C. albicans* infection, mice were euthanized, and cells from lymph nodes were stained for flow cytometry analysis. *n* = 15. (*M*) *C. albicans* were cultured in vitro in the presence of DMSO, AGK2 (10 μM), or AK-1 (10 μM) for the indicated times, and optical density (OD) values were measured at the indicated time points. **P < 0.01; ***P < 0.001; ****P < 0.0001 based on two-tailed unpaired Student’s *t* test (*A*, *B*, *E*, *F*, *I*, *J*, *K*, and *L*), two-way ANOVA (*D* and *H*), and log-rank (Mantel–Cox) test (*C* and *G*). NS: no significance. All error bars represent SEM of technical replicates. Data are pooled from three independent experiments.

Given our finding that the acetylation of α-tubulin is essential for the activation of antifungal signaling, we hypothesized that enhanced α-tubulin acetylation might augment antifungal immune responses at the cellular level. We therefore explored whether deacetylase inhibitors have any impact on antifungal immune signaling at the cellular level. Indeed, pretreatment of BMDMs with either the deacetylase Sirt2 or the HDAC6 inhibitors AGK2 or Tubastatin resulted in significantly increased proinflammatory gene expression following curdlan or α-mannan stimulation. Notably, AGK2 had a significantly greater effect than Tubastatin A on the increase in proinflammatory gene expression (Fig. 7 *A* and *B*). Again, utilizing the in vivo model of invasive candidiasis, we found that AGK2 administered 12 h after *C. albicans* infection resulted in significantly prolonged survival compared to treatment with diluent alone (Fig. 7*C*). We also observed decreased weight loss and reduced fungal burden in the kidneys of mice treated with AGK2 (Fig. 7 *D* and *E*). Histopathological analysis also confirmed the reduction in fungal burden in the kidneys of mice treated with AGK2 (Fig. 7*F*). Pretreatment of BMDMs with another Sirt2 inhibitor, AK-1, also resulted in increased proinflammatory gene expression after Dectin ligand stimulation (*SI Appendix*, Fig. S6*A*). Small interfering RNA (siRNA knockdown of Sirt2 in BMDMs led to increased proinflammatory gene expression compared to control siRNA transfection after curdlan or α-mannan stimulation, and these data indicate that Sirt2 negatively regulates antifungal immunity, most likely through the deacetylation of α-tubulin (*SI Appendix*, Fig. S6 *B*–*D*). The administration of AK-1 after *C. albicans* infection also resulted in prolonged survival, decreased weight loss, and decreased fungal burden in the kidney compared to control mice (Fig. 7 *G*–*I*). Interestingly, AK-1 significantly promoted the infiltration of macrophages and neutrophils into the kidneys of fungi-infected mice (Fig. 7 *J* and *K*). The Sirt2 inhibitor AK-1 also significantly increased Th17 cell differentiation in the lymph nodes compared to the control treatment (Fig. 7*L*). To exclude the possibility that the in vivo effect of AGK2 and AK-1 was due to direct growth inhibition of *C. albicans*, we examined whether AGK2 and AK-1 can directly inhibit fungal growth. We did not find that AGK2 and AK-1 had any inhibitory effect on the growth of *C. albicans* (Fig. 7*M*). Together, these data indicate that the deacetylase Sirt2 is a therapeutic target for the treatment of systemic fungal sepsis, and inhibitors of Sirt2 have the potential to be developed as drugs for the treatment of invasive fungal infection.

### The Deacetylase Sirt2 Inhibitor AK-7 Has a Therapeutic Effect on CNS Fungal Infection.

The CNS is commonly invaded by fungal species, and fungal infections of the CNS have high mortality rates of over 50% (6, 7). Fungal infections of the CNS are challenging to treat due to impaired drug penetration (62). It was reported that microglia in the brain play a critical role in CNS antifungal innate immunity by producing IL-1β and CXCL1 and subsequently recruiting neutrophils (6). We first examined whether Myo1f also plays any role in the antifungal immune response in microglia. The induction of the expression of proinflammatory genes, such as *IL-1β* and *CXCL1*, was almost abolished in Myo1f-KO microglia compared to control microglial cells after HKCA or α-mannan stimulation, which indicates that Myo1f also plays an essential role in the antifungal immune response in microglia (*SI Appendix*, Fig. S7*A*). Interestingly, we did not detect *IL-12A* or *IL-23A* expression in microglia, which was different from what we observed in BMDMs. As there is a Sirt2 inhibitor, AK-7, which can specifically cross the blood–brain barrier (63, 64), we next examined whether AK-7 has any therapeutic effect on CNS fungal infection. AK-7 increased HKCA*-* or α-mannan–induced proinflammatory gene expression in primary mouse microglial cells, and the administration of AK-7 by intraperitoneal injection after *C. albicans* infection significantly decreased the fungal burden in the mouse brain (*SI Appendix*, Fig. S7 *B* and *C*). Neutrophil recruitment was significantly increased in the brains of AK-7–treated mice compared to control mice (*SI Appendix*, Fig. S7*D*), which was consistent with a previous report that neutrophils were recruited into the brain to eliminate fungal infection (6). The recruitment of macrophages and the expansion of macroglia in the brain were also significantly increased after AK-7 treatment (*SI Appendix*, Fig. S7*D*). These data indicate that Myo1f also plays a critical role in the CNS antifungal immune response, and the CNS-permeable Sirt2 inhibitor AK-7 increases CNS antifungal immunity by promoting inflammatory gene expression in microglia and promoting the recruitment of neutrophils and macrophages into the CNS for the elimination of fungi.

### AGK-2 Promotes Proinflammatory Gene Expression in Human Peripheral Blood Mononuclear Cells after Dectin Stimulation.

To further confirm the role of MYO1F in human antifungal immunity, we knocked down the expression of MYO1F in human peripheral blood mononuclear cells (PBMCs) isolated from the peripheral blood of healthy donors. Importantly, knockdown of MYO1F expression in human PBMCs significantly reduced the proinflammatory cytokine expression induced by HKCA or α-mannan (*SI Appendix*, Fig. S8 *A* and *B*). These data further confirmed our early data from THP-1 cells and mouse BMDMs (Figs. 1 *A* and *B* and 2 *A*–*H*). Consistent with the data from mouse BMDMs, AGK2 significantly promoted proinflammatory gene expression compared to control dimethyl sulfoxide (DMSO) treatment after HKCA or α-mannan stimulation (*SI Appendix*, Fig. S8*C*). These data indicate that MYO1F plays a critical role in human antifungal immunity and that inhibitors of Sirt2 have the potential to be drugs for the treatment of fungal infections.

## Discussion

Previous studies found that MYO1F was mainly involved in the regulation of immune cell adhesion, motility, and migration (30–32, 34), while few studies have identified a functional role for this molecule in innate immune signaling pathways, such as the TLR and CLR signaling pathways. In this study, we show that MYO1F plays a critical role in the activation of antifungal innate immune signaling. Mechanistically, MYO1F functions as an adaptor by recruiting the AP2A1/αTAT1 acetyltransferase complex to α-tubulin and thereby promotes the acetylation of α-tubulin; then, acetylated α-tubulin plays a critical role in the translocation of the key signaling molecules Syk and CARD9 from the cell membrane to the cytoplasm, which is an essential step in antifungal signal transduction (*SI Appendix*, Fig. S8*D*). Notably, we report that inhibitors of the Sirt2 deacetylase, AGK2, AK-1, or AK-7 increase proinflammatory gene expression in response to dectin ligands and protect mice from the lethal sequelae of systemic or CNS *C. albicans* infection. Taken together, these results suggest that Sirt2 represents a potential target for antifungal drug development, and inhibitors of Sirt2 may have potential use as a therapy for patients with invasive fungal infection.

Many previous studies have established that signaling molecules such as Syk are capable of translocating to the cell membrane in response to membrane receptor activation by fungal stimuli (16, 65). While the translocation of Syk and CARD9 from the membrane to the cytoplasm has not been previously characterized, our results indicate that the loss of Syk and CARD9 translocation from the membrane to the cytoplasm abrogates antifungal signal transduction. It therefore seems possible that the trafficking of signaling proteins from the membrane to the cytoplasm following membrane-proximal signaling may represent a critical component of downstream signal transduction and cellular activation. This “membrane to cytoplasm trafficking” may be universal to many other signaling pathways and has been neglected in most cases in the past; thus, this pathway needs to be further investigated in the future. Interestingly, it seems that MYO1F only regulates the acetylation of membrane-localized α-tubulin but not centromeric acetylated α-tubulin (Fig. 5 *B* and *C*); these findings are consistent with a previous report that the acetylase αTAT1 localizes to CCPs beneath the cellular membrane through a direct interaction with AP2A1, which is required for microtubule acetylation, and this AP2A1-αTAT1–mediated α-tubulin acetylation accounts for only approximately one-third of growing microtubules in the cells (57). The acetylation of microtubules in CCPs plays a critical role in cell migration and invasion, which could be the reason that previous studies have found that MYO1F-KO cells show defective motility and migration (30, 31, 34). Acetylated α-tubulin also mediates inflammasome activation by transporting ASC on mitochondria to NLRP3 on the endoplasmic reticulum (33, 47). Therefore, the signaling molecule transport between mitochondria and the endoplasmic reticulum may also be dependent on MYO1F-mediated α-tubulin acetylation, and this needs to be further elucidated in the future. Another interesting topic is identifying the mechanism by which fungi induce α-tubulin acetylation and determining whether this mechanism is regulated by the decreased cellular level of NAD+, which leads to the inactivation of Sirt2 as found in the process of NLRP3 inflammasome activation (47). In addition to regulating antifungal immunity in the macrophages, we also found that Myo1f may regulate B cell development, as B cell abundance was significantly reduced in the peripheral blood and spleens of Myo1f-KO mice compared to WT mice (*SI Appendix*, Fig. S5 *A* and *B*). These findings suggest that Myo1f may regulate mouse B cell development that might further play a role in the observed antifungal immunity defects in Myo1f-KO mice as recently demonstrated by Itai Doron and colleagues that host protective antifungal antibodies play an indispensable role in the antifungal immunity (58). This point needs to be further investigated in the future.

At this writing, there are limited antifungal therapies approved for the treatment of invasive fungal infections, and in particular, the treatment of CNS fungal infection represents a substantial challenge due to impaired drug penetration into the CNS and resistance to antifungal drug treatments (2, 62). The impact of fungal infection on human health has nearly reached crisis proportions (2). Thus, an improved understanding of the mechanisms that promote host defense against pathogenic fungi is important. It will be critical to understand how our immune system recognizes and distinguishes among fungi, which may also provide useful methods and targets for the treatment of human fungal infection. Our study identified the deacetylase Sirt2 as a negative regulator of antifungal immunity (*SI Appendix*, Fig. S6 *B*–*D*), and its inhibitors, AGK2, AK-1, and AK-7, are agents that both increased antifungal signaling and protected mice from both systemic and CNS *C. albicans* infection (Fig. 7 and *SI Appendix*, Fig. S7). These results suggest that Sirt2 inhibitors have potential as therapeutic agents for the treatment of fungal infections. Our study also notes that the deacetylase Sirt2 is a therapeutic target for antifungal drug development. Since multiple Sirt2 inhibitors have been developed for the treatment of cancers or neurodegenerative diseases (66–69), it could be important to test whether these existing Sirt2 inhibitors have any potential as antifungal drugs.

## Materials and Methods

### Human PBMCs Study.

Whole blood samples were drawn from each study participant. Human PBMCs were isolated by Ficoll-paque PREMIUM (17-5442-02, GE Healthcare) from freshly drawn peripheral venous blood from healthy controls according to manufactory instruction. Briefly, we added Ficoll-Paque media to the centrifuge tube and layered the diluted blood sample onto the Ficoll-Paque media solution. We then centrifuged at 400 *g* for 30 to 40 min at 18 to 20 °C with the brake turned off. Then, we drew off the upper layer containing plasma and platelets using a sterile pipette, leaving the mononuclear cell layer undisturbed at the interface. Finally, we washed the layer of mononuclear cells twice with 1× phosphate-buffered saline.

For the siRNA knockdown assays, human PBMCs were transfected with Rfect^SP^ siRNA Transfection Reagent (Changzhou Bio-generating Biotechnology Corp) according to manufactures’ instruction. Human PBMCs were stimulated with HKCA (multiplicity of infection [MOI] = 2) or α-mannan (100 μg/mL) 5 d after transfection and harvested for gene expression analysis.

For the stimulation of human PBMCs, cells were pretreated with DMSO or AGK-2 (10 μM) for 1 h at 37 °C before being stimulated with HKCA*s* (MOI = 2) or α-mannan (100 μg/mL). Cells were then harvested for gene expression. This study followed the guidelines set forth by the Declaration of Helsinki, and the protocol passed the review of the Ethics Committee of Tongji Medical College, Huazhong University of Science and Technology. All study participants have signed a written informed consent form.

#### Mice.

The Myo1f-KO mouse was made by Cyagen Biosciences, Inc. by CRISPR-Cas9 technique as demonstrated in *SI Appendix*, Fig. S1*B*. A 5,077–base pair DNA fragment was depleted by these two guide RNAs, which was confirmed by genotyping and Western blot analysis. To avoid possible off-target editing of the mice genome, the mice were bred six generation with mice of a C57BL/6 background before performing any experiments. Mice were housed under specific pathogen-free conditions, and the experimental protocols were approved by the Institutional Animal Care and Use Committee of the Huazhong University of Science and Technology.

#### Cells.

293T and HeLa cells were maintained in Dulbecco’s Modified Eagle Medium plus 10% fetal bovine serum (FBS) and 1% Penicillin-Streptomycin. THP-1 cells were maintained in Roswell Park Memorial Institute 1640 plus 10% FBS and 1% Penicillin-Streptomycin and 1% sodium pyruvate.

#### Statistics.

The statistical significance between two groups was determined by unpaired two-tailed Student’s *t* test; multiple-group comparisons were performed using one-way ANOVA; and the clinical scores and weight change curve were analyzed by two-way ANOVA for multiple comparisons. *P <* 0.05 was considered to be significant. The results are shown as mean, and the error bar represents SE of mean biological or technical replicates as indicated in the figure legend.

## Supplementary Material

Supplementary File

Supplementary File

Supplementary File

Supplementary File

Supplementary File

Supplementary File

Supplementary File

## Data Availability

The mass spectrometry data are in Datasets S1–S6 and were deposited into public database “figshare,” and the access links are https://figshare.com/articles/dataset/TAGAP_MS_zip/14446296 and https://figshare.com/articles/dataset/MYO1F_MS_zip/14447094. All other study data are included in the article and *SI Appendix*.
