# Supplementary material for: MYO1F regulates antifungal immunity by regulating acetylation of microtubules
Source: Proc Natl Acad Sci U S A. 2021 Jul 23;118(30):e2100230118. doi: 10.1073/pnas.2100230118 (PMC8325298; doi:10.1073/pnas.2100230118)
Supplement: Supplementary File [file pnas.2100230118.sapp.pdf]

Supplementary Information for

MYO1F regulates anti-fungal immunity by regulating acetylation of microtubules

Wanwei Sun[1,9*], Xiaojian Ma[1,9], Heping Wang[1], Yanyun Du[1], Jianwen Chen[1], Huijun Hu[1], Ru Gao[1], Ruirui He[1], Qianwen Peng[1], Zhihui Cui[1], Huazhi Zhang[1], Junhan Wang[2], Xinming Jia[3], Bradley N. Martin[4], Cun-Jin Zhang[5*], Xiaoxia Li[6], Chenhui Wang[1,7,8*]

1 Key Laboratory of Molecular Biophysics of the Ministry of Education, National Engineering Research Center for Nanomedicine, College of Life Science and Technology, Huazhong University of Science and Technology, Wuhan 430074, China.

2 University-Affiliated Hospital, Huazhong University of Science and Technology, Wuhan 430074, China.

3 Shanghai Skin Disease Hospital, Tongji University School of Medicine, Shanghai, China.

4 Department of Medicine, Brigham and Women's Hospital, Harvard Medical School, Boston, USA.

5 Department of Neurology of Nanjing Drum Tower Hospital, Medical School and the State Key Laboratory of Pharmaceutical Biotechnology, Nanjing University, Nanjing, Jiangsu 210008, China.

6 Department of Inflammation and Immunity, Lerner Research Institute, Cleveland Clinic, Cleveland, OH, 44106, USA.

7 Wuhan Institute of Biotechnology, Wuhan 430070, China.

8 Lead contact

9 Equal contribution: Wanwei Sun and Xiaojian Ma

* Correspondence: Wanwei Sun, Cun-jin Zhang and Chenhui Wang

Email: 2018501003@hust.edu.cn, zhangcj@nju.edu.cn and wangchenhui@hust.edu.cn

This PDF file includes:

SI Materials and Methods

Figures S1 to S8

Legends for dataset S1-S6

Materials and methods

Reagents. Antibodies of anti-Syk (clone D3Z1E), anti-p-IκBα (clone 14D4), anti-p-p38 (clone D3F9), anti-p-JNK (clone 81E11), anti-HA (clone C29F4), anti-Flag (clone D6W5B) and anti-α-Tubulin (clone 11H10) were bought from Cell Signaling Technology (cat no. 13198, 2859, 4511, 3724, 14793 and 2125). Antibodies of anti-HA (clone HA-7) and anti-Flag (clone M2) antibodies for immunoprecipitation were bought from Sigma (cat no. H9658 and F1804). Antibodies of anti-Myo1f (clone B-5), anti-IKKα (clone M-280), anti-CARD9 (clone A-8), anti-p-ERK (clone 12D4), anti-p-Tyrosine (clone PY20), anti-Dynein (clone 74-1), anti-HSP90 (clone AC-16), anti-Actin (clone C-2) and anti-GAPDH (clone 2E3-2E10) were bought from SANTA CRUZ BIOTECHNOLOGY (cat no. sc-376534, sc-7182, sc-374569, sc-81492, sc-508, sc-13524, sc-130068, sc-8432 and sc-293335). Antibody of anti-AP2A1, anti-IKKβ, anti-Sirt2, anti-Syk, anti-ERK, anti-p38 and anti-JNK were bought from Abclonal (cat no. A6863, A2087, A0273, A2123, A16686, A14401 and A18287). Antibody of anti-Ac-α-Tubulin (clone 6-11B-1) was bought from Sigma (cat no: T7451). Flow antibodies of anti-CD45 (clone 30-F11), anti-Ly6G (clone 1A8), anti-F4/80 (clone BM8) and anti-CD11b (clone M1/70) were bought from Biolegend (cat no: 103108, 127608, 123115 and 101212). Zombie Violet Fixable Viability kit was bought from Biolegend (cat no. 423114). Zymosan, D-zymosan, curdlan and LTA were bought from Invivogen (cat no. tlrl-zyn, tlrl-zyd, tlrl-cud and tlrl-pslta). α-mannan was bought from Sigma (cat no. M3640). Colchicine, Nocodazole, AGK2 and Tubastatin-A were bought from Topscience (cat no. T0320, T2802, T6371 and T1966). AK-1 and AK-7 were bought from MCE (HY-101465 and HY-16691). Ciliobrevin-D was bought from Sigma (cat no. 250401). Recombinant mouse M-CSF proteins were bought from Peprotech (cat no. 315-02).

Immunofluorescence. Cells were fixed with 4% paraformaldehyde and followed by permeabilization treatment with PBS containing 0.3% Triton X-100 for 10 minutes. Prior to incubation with primary antibody, samples were incubated with 10% goat serum at room temperature for 1 hour to block non-specific staining. After 12 hours of incubation with primary antibody at 4°C, the samples were washed three times with ice cold PBS and further stained with fluorophore (Alex 488 or Alex 405 and Alexa 594 conjugated secondary antibodies). After staining, samples were counter stained with DAPI and immersed in mounting medium before being sealed on a slide with nail polish. Sealed slides were analyzed using OLYMPUS FV3000 microscope with companion software. For the calculation of cell percentage with co-localization of Syk and CARD9, control cells or MYO1F-KO THP-1 cells were left untreated or stimulated with heat-killed *C. albicans* (MOI=2) for 30 and 60 mins, followed by immunofluorescence analysis by the confocal microscope. 3×300 cells each time were analyzed under confocal microscope (300 cells each time, and total 3 times), and the percentage of cells with co-localization of Syk and CARD9 / total cells analyzed was shown.

Mouse BMDMs preparation. BMDMs were obtained by differentiating bone marrow progenitors from the tibia and femur of 6–8-week-old male or female mice in Iscove's Modified Dulbecco's Media (IMDM) containing 20 ng/ml of M-CSF, 10% heat-inactivated fetal bovine serum (FBS, Invitrogen), 1 mM sodium pyruvate, 100 U/ml penicillin, and 100 μg/ml streptomycin (Invitrogen) for 5-7 days.

Cells were then re-plated in 6-well or 12-well plates 1 day before experiments.

Primary mouse microglia isolation. Primary microglia were obtained by from 6–8-week-old naïve male or female mice. Briefly, brains were homogenized in ice cold tissue grinders, filtered through a 100 µm cell strainer and the cells collected by centrifugation at 400g for 5 min at 4 °C. Cells were resuspended in 10ml of 30% Percoll (Amersham Bioscience) and centrifuge onto a 70% Percoll cushion in 15-ml tubes at 800g for 30 min. Cells at the 30–70% interface were collected and were subjected to flow cytometry sorting according to the sorting strategy manifested in the Supplemental Figure 7D.

SiRNA knockdown protocol. BMDMs were obtained by differentiating bone marrow progenitors from the tibia and femur of 6–8-week-old male or female mice in Iscove's Modified Dulbecco's Media (IMDM) containing 20 ng/ml of M-CSF, 10% heat-inactivated fetal bovine serum (FBS, Invitrogen), 1 mM sodium pyruvate, 100 U/ml penicillin, and 100 µg/ml streptomycin (Invitrogen) for 5 days. Cells were transfected with GenMute^{TM} siRNA Transfection Reagent (SignaGen Laboratories) according to manufactures' instruction. 5 days after transfection, mouse primary macrophages were harvested for immunoblot and Real-time PCR.

Immunoblot and immunoprecipitation. Cell were harvested and lysed on ice in lysis buffer which containing 0.5% Triton X-100, 20 mM Hepes pH 7.4, 150 mM NaCl, 12.5 mM β-glycerophosphate, 1.5 mM MgCl2, 10 mM NaF, 2 mM dithiothreitol, 1 mM sodium orthovanadate, 2 mM EGTA, 20 mM aprotinin, and 1 mM phenylmethylsulfonyl fluoride for 30 minutes, followed by centrifuging at 12,000 rpm for 15 minutes to extract clear lysates. For immunoprecipitation, cell lysates were incubated with 1 µg of antibody at 4 degree overnight, followed by incubation with A-sepharose or G-sepharose beads for 2 hours, and the beads were washed four times with lysis buffer and the precipitates were eluted with 2× sample buffer. Elutes and whole cell extracts were resolved on SDS-PAGE followed by immunoblotting with antibodies.

RT and Real-time PCR. Total RNA was extracted from spinal cord with TRIzol according to the manufacturer's instructions. 1 µg total RNA for each sample was reverse transcribed using the SuperScript® II Reverse Transcriptase from Thermo Fisher Scientific. The resulting complementary DNA was analyzed by real-time PCR using SYBR Green Real-Time PCR Master Mix. All gene expression results were expressed as arbitrary units relative to expression *Actb* or *GAPDH*.

Lentivirus-medicated gene knockout/knockdown in THP-1 cells. pLentiCRISPR-GFP vector was used for CRISPR/Cas9-mediated gene knockout in THP-1 cell line. Briefly, lentivirus vector expressing gRNA was transfected together with package vectors into HEK293T (ATCC) package cells. 48 and 72 hours after transfection, virus supernatants were harvested and filtrated with 0.2 µm filter. Target cells were infected twice and sorted by flow cytometry medicated cell sorting. For some experiments, single cell was plated into 96-well plate by flow cytometry for single clone isolation. Isolated single clones were verified by western blot and DNA sequencing.

Flow cytometry for brain infiltrated cells. One days after live *C. albicans* infection, mice were sacrificed and perfused with 1×PBS. Brains were homogenized in ice cold tissue grinders, filtered

through a 100 μm cell strainer and the cells collected by centrifugation at 400g for 5min at 4 °C. Cells were resuspended in 10ml of 30% Percoll (Amersham Bioscience) and centrifuge onto a 70% Percoll cushion in 15-ml tubes at 800g for 30min. Cells at the 30–70% interface were collected and were subjected to flow cytometry. Cell surface staining was done for 30 min at 4°C. Zombie Violet Fixable Viability kit (1:400; Biolegend) was added to exclude dead cells. Flow cytometry data analysis was performed by using CytExpert. General flow cytometry gating strategy was shown in Supplemental Figure 7D.

Bone marrow–chimeric mice. Six-week-old recipient mice were lethally irradiated by X-ray (550 rad×2), and 5×10^6 BM leukocytes from donors were intravenously transferred to the mice. Chimeras were used for further experiments 7 weeks after the initial reconstitution.

BMDMs phagocytosis assay and ROS production assay. For BMDMs phagocytosis assays, *C. albicans* (strain SC5314) cells were labeled with Alexa Fluor 488 in 100 mM HEPES buffer (pH 7.5). BMDMs were co-cultured with labeled *C. albicans* at 37 °C for 45 mins. Fluorescence signals from fungal cells that were adherent to, but not phagocytosed by, phagocytes were quenched with trypan blue, and the rate of phagocytosis was assessed by flow cytometry. For GFP-*C. albicans*, funguses were fixed by 2% paraformaldehyde at room temperature for 30 mins, then were co-cultured with BMDMs for the indicated times. Unbound yeasts were gently washed by 1×PBS with five times, cells were analyzed by flow cytometry.

For ROS production assay, ROS was measured by DHE (Dihydroethidium) fluorescent probe according to the manufacturer's instructions. Briefly, 2×10^5 wild-type THP-1 cells or MYO1F-KO THP-1 cells were differentiated by PMA for 2 days and then were washed with PBS twice. DHE was added to fresh medium at the final concentration of 1 μM and cells were incubated at 37°C for 30 minutes before stimulated with heat-killed *C. albicans* (MOI=2) or α-mannan (100 μg/ml). ROS production was determined by flow cytometry.

Fungal killing assay. For *in vitro* fungal killing assay, differentiated THP-1 cells or MYO1F -KO THP-1 cells (5×10^5/well) were incubated with *C. albicans* (MOI=10) for 24h. After co-culture, cells were washed by 1×PBS with three times, then resuspended in fresh medium containing Amphotericin B (Sigma Aldrich, V900919) at a final concentration of 30 μg/ml and cultured for 3h at 37ºC. Cells were washed three times in PBS again, then lysed by the addition of 0.02% Triton X-100 (Sigma Aldrich, T8787) and 100 μl suspension was spread (1:1000 dilution) on YPD plates. After incubation at 37 °C for 24h, killing was determined by counting the *Candida* colonies.

Mass spectrometry identification of MYO1F-interacting proteins. For the identification of MYO1F-interacting proteins, PCDH-EGFP-puro empty vector or PCDH-EGFP-puro-Flag-hMYO1F stable infected THP-1 cells were differentiated with PMA (25 ng/mL) for 3 days, followed by immunoprecipitation by anti-Flag antibody. After washing for four times with IP buffer, protein was eluted with IP buffer containing 0.1% of SDS and analyzed by mass spectrometry analysis.

Samples were reduced and alkylated in dithiothreitol (DTT) and iodoacetamide followed by trypsin digestion overnight. Digested samples were injected onto Agilent Zorbax 300SB-C18

0.075mm×150mm column on Eskigent nano LC system coupled with Thermo LTQ-ETD-Orbitrap. Advion Triversa nanomate served as the nano-ion spray source. MSMS data were searched against Refseq human protein database by Sorcerer Sequest. The searched dataset was processed by TPP (Trans-Proteomics Pipeline) and filtered with Peptide Prophet.

The raw data for the mass spectrometry identification of TAGAP- or MYO1F-interacting were deposited in the public database "figshare", and the access link are https://figshare.com/articles/dataset/TAGAP_MS_zip/14446296 and https://figshare.com/articles/dataset/MYO1F_MS_zip/14447094.

Histopathology. For histopathology analyses, kidneys were fixed in 10% neutral-buffered formalin, processed according to standard procedures, embedded in paraffin, and sectioned. 2-µm-thick sections were stained with hematoxylin and eosin (H&E), periodic-acid-Schiff (PAS). Renal inflammation was scored based upon H&E and PAS staining (proportion of renal parenchyma and/or pelvis involved by tubulointerstitial nephritis and/or pyelonephritis) as not significant (score 0), less than 10% (score 1), 10–25% (score 2), 25–50% (score 3), or greater than 50% (score 4). The intra-lesional fungal burden was based upon PAS staining as not significant (score 0), scant presence in less than 10% of inflammatory foci (score 1), mild-to-moderate presence in 10–25% of inflammatory foci (score 2), moderate-to-significant presence in 25–50% of inflammatory foci (score 3), or significant presence in more than 50% of inflammatory foci (score 4).

Systemic or CNS *C. albicans* infection model. Live *C. albicans* strain SC 5314 (4×10^5 yeast cells in 0.1 ml of 1×PBS buffer) were injected intravenously into 6- to 8-week-old littermates of distinct genotypes. Infected mice were monitored daily for weight loss and survival. Fungal burden of kidneys was measured 2 days after infection. After the kidneys were collected, tissue homogenates were serially diluted and plated on yeast extract–peptone–dextrose agar. Fungal colony-forming units were counted 24 hours after plating. For the CNS *C. albicans* infection model, 2×10^5 live *C. albicans* strain SC 5314 yeast cells in 0.1 ml of 1×PBS buffer were injected intravenously into 6- to 8-week-old WT mice, and two days after infection, the mice were sacrificed and perfused with 1×PBS. Mice brains were collected, and tissue homogenates were serially diluted and plated on yeast extract–peptone–dextrose agar. Fungal colony-forming units were counted 24 hours after plating.

For the treatment of AGK-2, AK-1 and AK-7 experiments, AGK-2 (10 µg/mouse), AK-1 (50 µg/mouse) or AK-7 (50 µg/mouse) was intraperitoneal injected 12 hours after *C. albicans* infection (intravenously injection of 2×10^5 live *C. albicans* strain SC 5314 yeast cells in 0.1 ml of 1×PBS), and weight loss and survival rate were calculated.

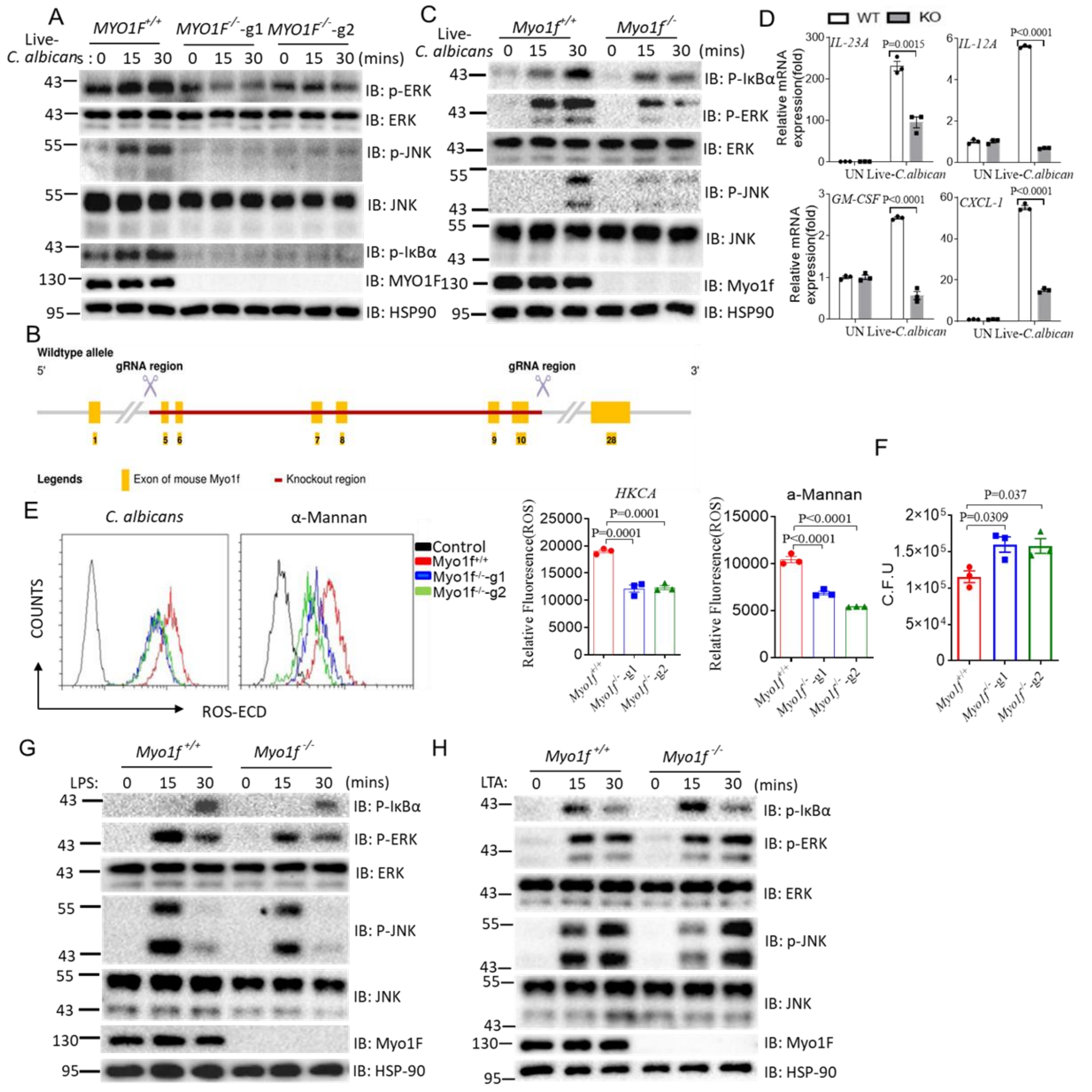

Figure S1. The design strategy of Myo1f-KO mice by Crispr-Cas9. (A) Wild-type control THP-1 cells or MYO1F-KO THP-1 cells were left untreated or stimulated with live *C. albicans* (MOI=1) for the indicated times, followed by western blot analysis of indicated proteins. **(B)** Sketch map of designation of Myo1f-KO mice by Crispr-cas9 technique was shown, and exons of no.5-no.10 was depleted by Crispr-Cas9 technique. **(C)** BMDMs from WT control mice or Myo1f-deficient mice were stimulated with live *C. albicans* (MOI=1) for the indicated time, followed by western blot analysis of indicated proteins expression. **(D)** BMDMs from WT control mice or Myo1f-deficient mice were stimulated with live *C. albicans* (MOI=1) for the indicated times, followed by RT and real-time PCR

analysis of indicated gene expression. **(E)** WT THP-1 cells or MYO1F KO THP-1 cells were stimulated with heat-killed *C. albicans* (MOI=2) or α-mannan (100 μg/ml) for 3 hours, followed by flow cytometry analysis of ROS generation. **(F)** Intracellular *C. albicans* survival was measured as described in the Methods. **(G-H)** BMDMs from WT control mice or Myo1f-deficient mice were stimulated with LPS (**G**, 100 μg/ml) or LTA (**H**, 100 μg/ml) for the indicated times, followed by western blot analysis of indicated proteins. *: P<0.05; **: P<0.01; ***: P<0.001; ****: P<0.0001 based on unpaired two-tailed t test (**D**), one-way ANOVA (**E** and **F**). Error bars represent S.E.M of biological replicates for **D**. Data are representative of three independent experiments.

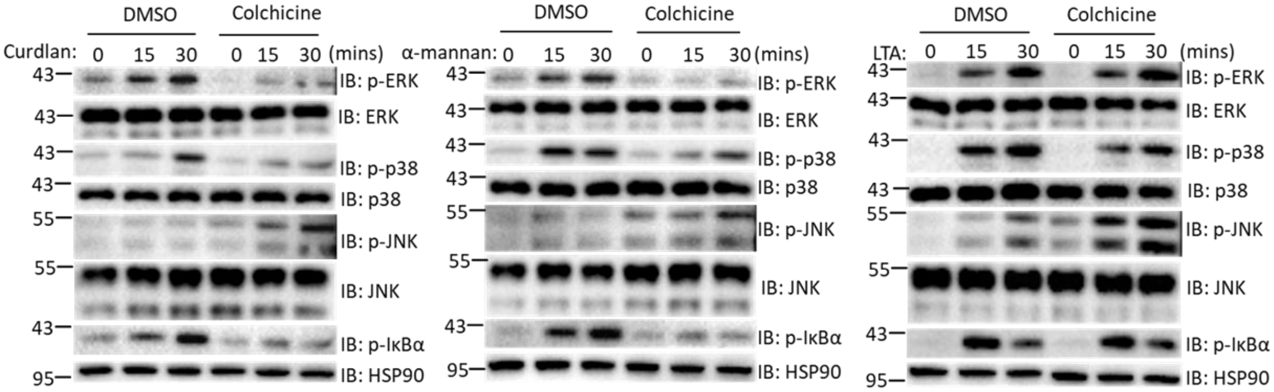

Figure S2. Colchicine inhibits the activation of antifungal signaling pathway. BMDMs from WT mice were pretreated with colchicine (10 μM) for 1 hour, followed by stimulation with Curdlan (100 μg/ml), α-mannan (100 μg/ml) or LTA (100 μg/ml) for the indicated times. Cell lysates were analyzed by western blot for indicated proteins. Data are representative of three independent experiments.

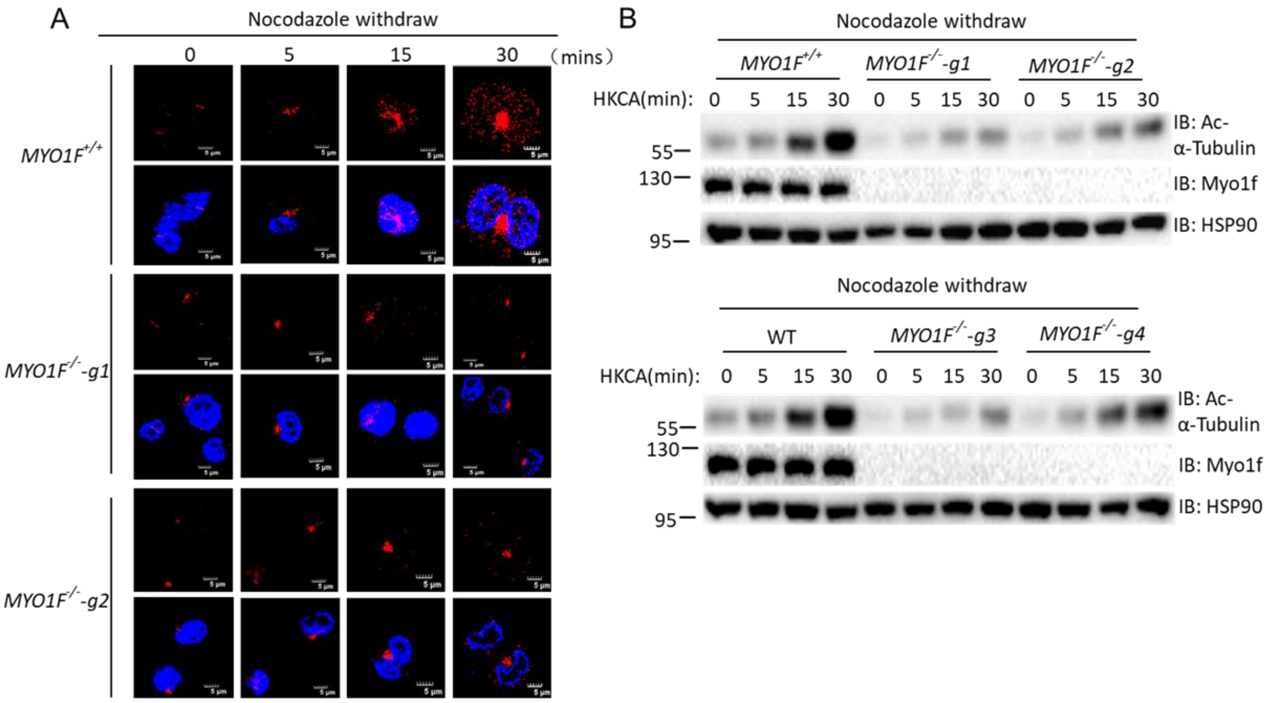

Figure S3. MYO1F is required for α-Tubulin acylation recovery after nocodazole washout. (**A**) WT or MYO1F-KO THP-1 cells were treated with nocodazole (10 μM) for 5 hours and then nocodazole was washed out for the indicated times, followed by immunofluorescence staining of indicated proteins. Scale bar=5 μm. (**B**) Wild-type control THP-1 cells or 4 different MYO1F-KO THP-1 cell clones were treated with nocodazole (10 μM) for 5 hours and then the drug was washed out for the indicated times, followed by western blot analysis of indicated proteins. Data are representative of three independent experiments.

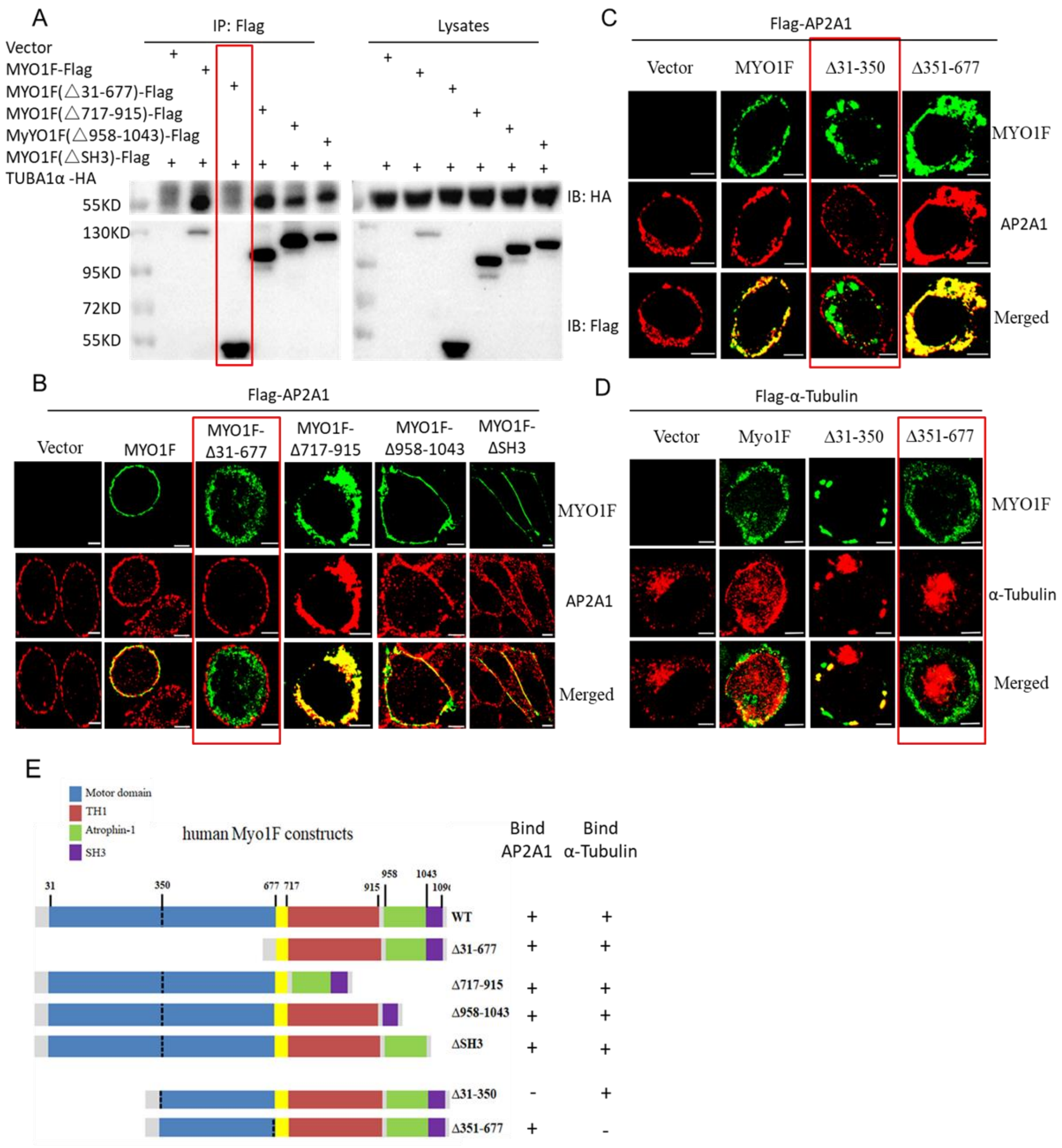

Figure S4. MYO1F binds to α-Tubulin and AP2A1 through different regions. (**A**) HEK293T cells were transfected with indicated plasmids, and cell lysates were immunoprecipitated with anti-Flag antibody, followed by immunoblot analysis for indicated proteins. **(B-D)** HEK293T cells were transfected with Flag-AP2A1 (**B-C**) or Flag-α-Tubulin (**D**) together with HA- MYO1F WT or different deletion mutants, followed by immunofluorescence staining of anti-Flag and anti-HA antibodies. Scale bar=5 μm. (**E**) Mapping sketch of MYO1F and AP2A1 or MYO1F and α-Tubulin was shown. Data are representative of three independent experiments.

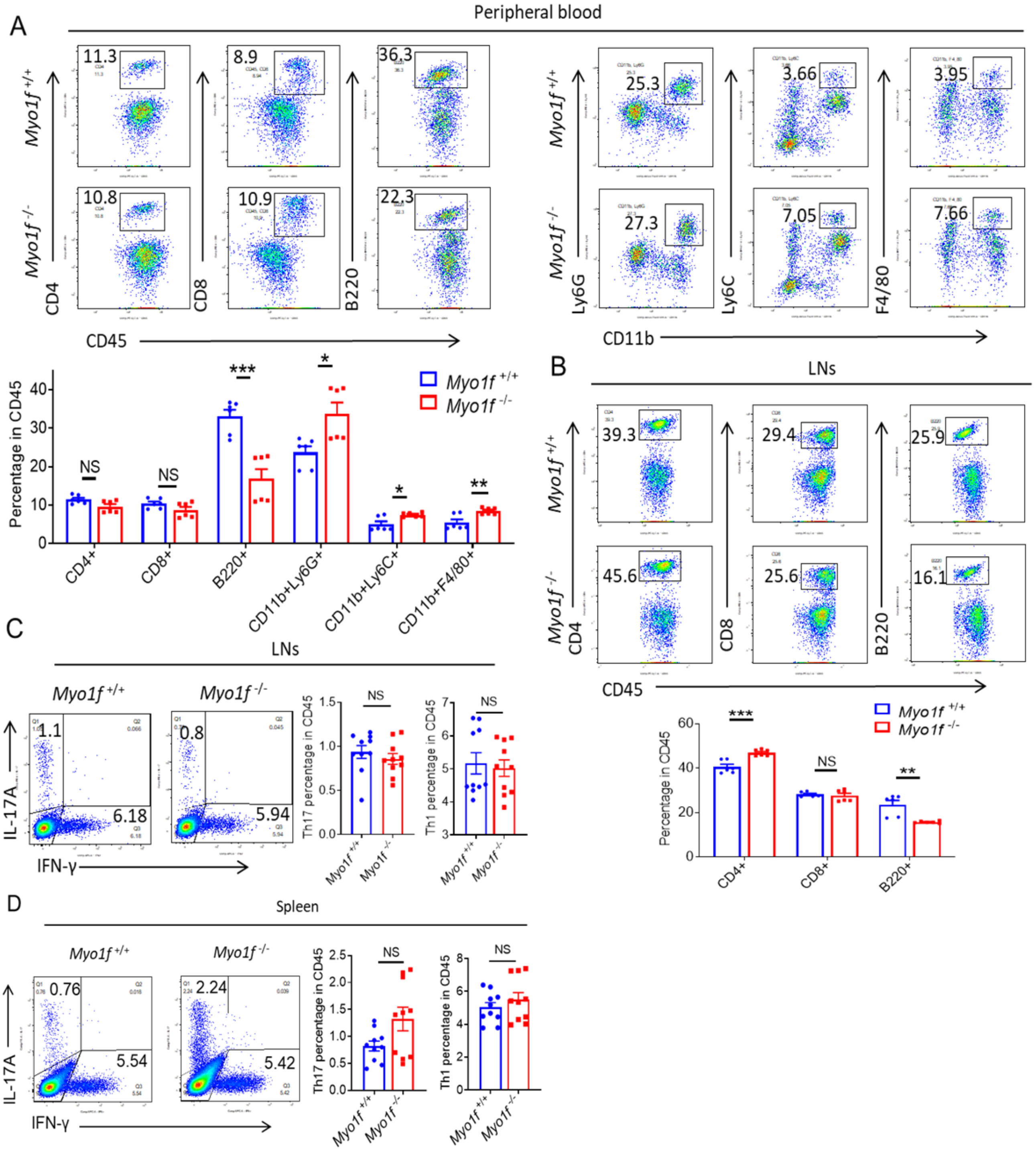

Figure S5. Characteristics of immune cells in the Myo1f-KO mice. (A-D) Cells were isolated from peripheral blood (**A**), lymph nodes (**B-C**) and spleens (**D**) of WT mice or Myo1f-KO mice, followed by flow cytometry analysis for the indicated immune cell populations. *: P<0.05; **: P<0.01; ***: P<0.001 based on unpaired two-tailed t test (**A-D**). n=6 for **A-B**, and n=10 for **C-D**. Error bars represent S.E.M of biological replicates. Data are representative of two independent experiments.

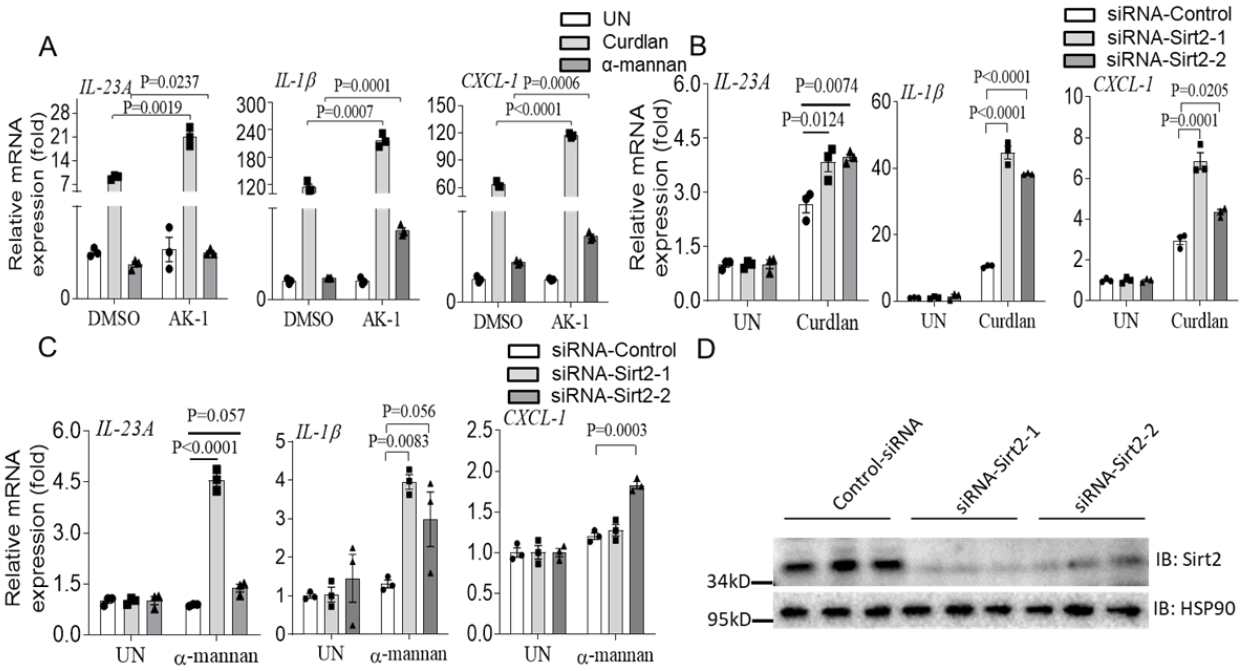

Figure S6. Knocking down of Sirt2 expression increases the antifungal immune response in BMDMs. (A) BMDMs from WT mice were pretreated with DMSO or AK-1 (10 μM) for one hour, and stimulated with Curdlan (100 μg/ml) or α-mannan (100 μg/ml) for 3 hours, followed by real-time PCR analysis of indicated gene expression. **(B-C)** WT BMDMs were transfected siRNA to knockdown the expression of Sirt2 as described in the METHODS, followed by stimulation with Curdlan (100 μg/ml, **B**) or α-mannan (100 μg/ml, **C**) for 3 hours. Gene expression was analyzed by real-time PCR analysis. (**D**) Western blot analysis of Sirt2 protein in control siRNA or Sirt2 siRNA transfected BMDMs was shown. All error bars represent S.E.M of technical replicates. *: P<0.05; **: P<0.01; ***: P<0.001 based on two-tailed unpaired t-test **(A)**, and one-way ANOVA for **B-C**. Data are representative of three independent experiments.

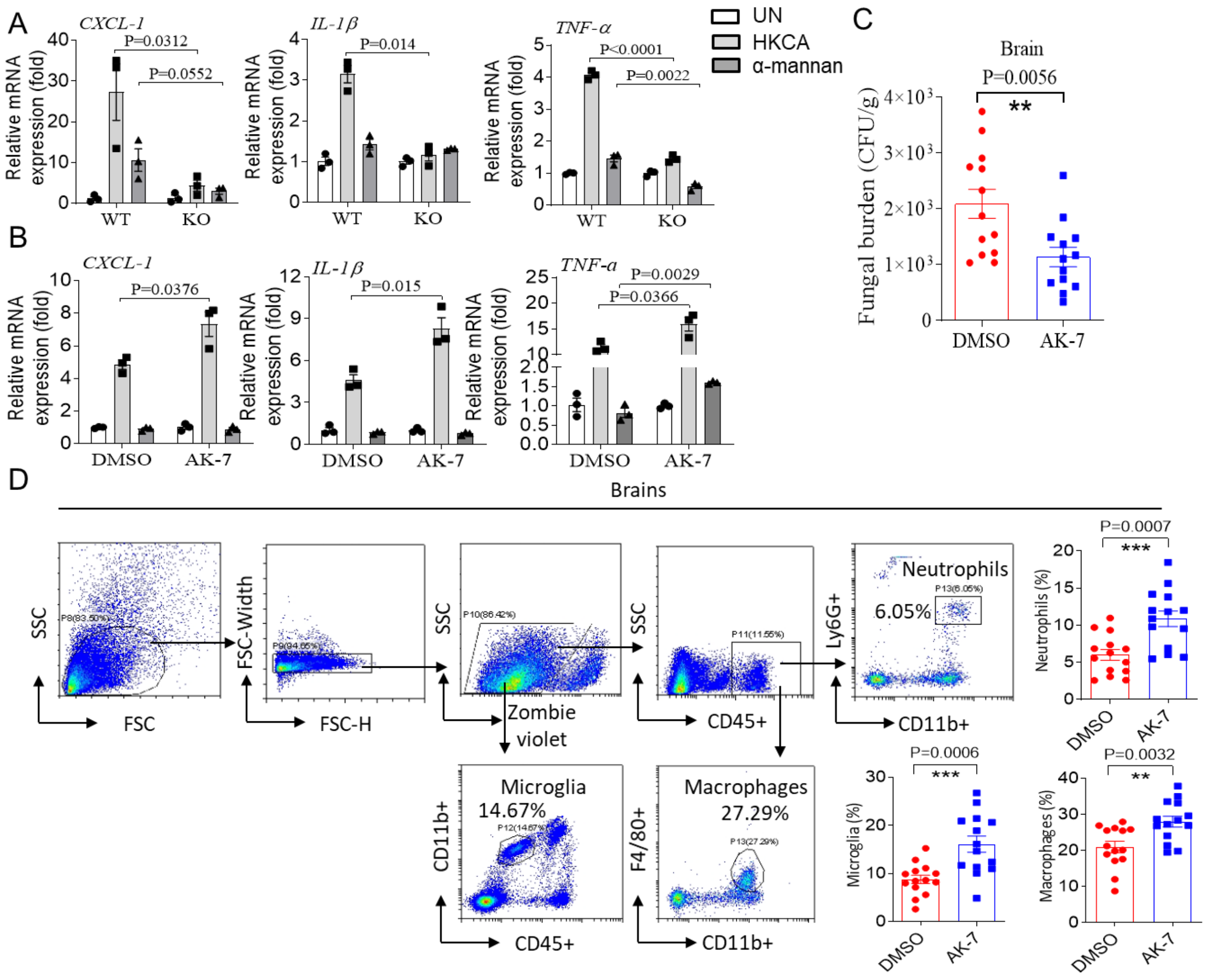

Figure S7. AK-7 has a therapeutic effect for the CNS fungal infection. (A) Primary microglia from WT mice or Myo1f-KO mice were stimulated with heat-killed *C. albicans* (HKCA, MOI=2) or α-mannan (100 μg/ml) for 3 hours, followed by real-time PCR analysis of indicated gene expression. (**B**) Primary WT microglia were pretreated with AK-7 (10 μM) for 1 hour, and then were stimulated with heat-killed *C. albicans* (HKCA, MOI=2) or α-mannan (100 μg/ml) for 3 hours, followed by real-time PCR analysis of indicated gene expression. (**C**) WT mice were intraperitoneal injected with DMSO or AK-7 (50 μg/mouse) 12 hours after intravenously injection of 2×10^5 live *C. albicans* yeast cells. Mice were sacrificed and brains were isolated 2 days after infection, and fungal burden in the brains were shown. n=13. (**D**) WT mice were treated as in **C**, and brain-infiltrated cells were isolated as described in the METHODS, and were analyzed by flow cytometry. n=14. *: P<0.05; **: P<0.01; ***: P<0.001 based on two-tailed unpaired t-test (**A-D**). Error bars represent S.E.M of biological replicates. Data are pooled from three independent experiments.

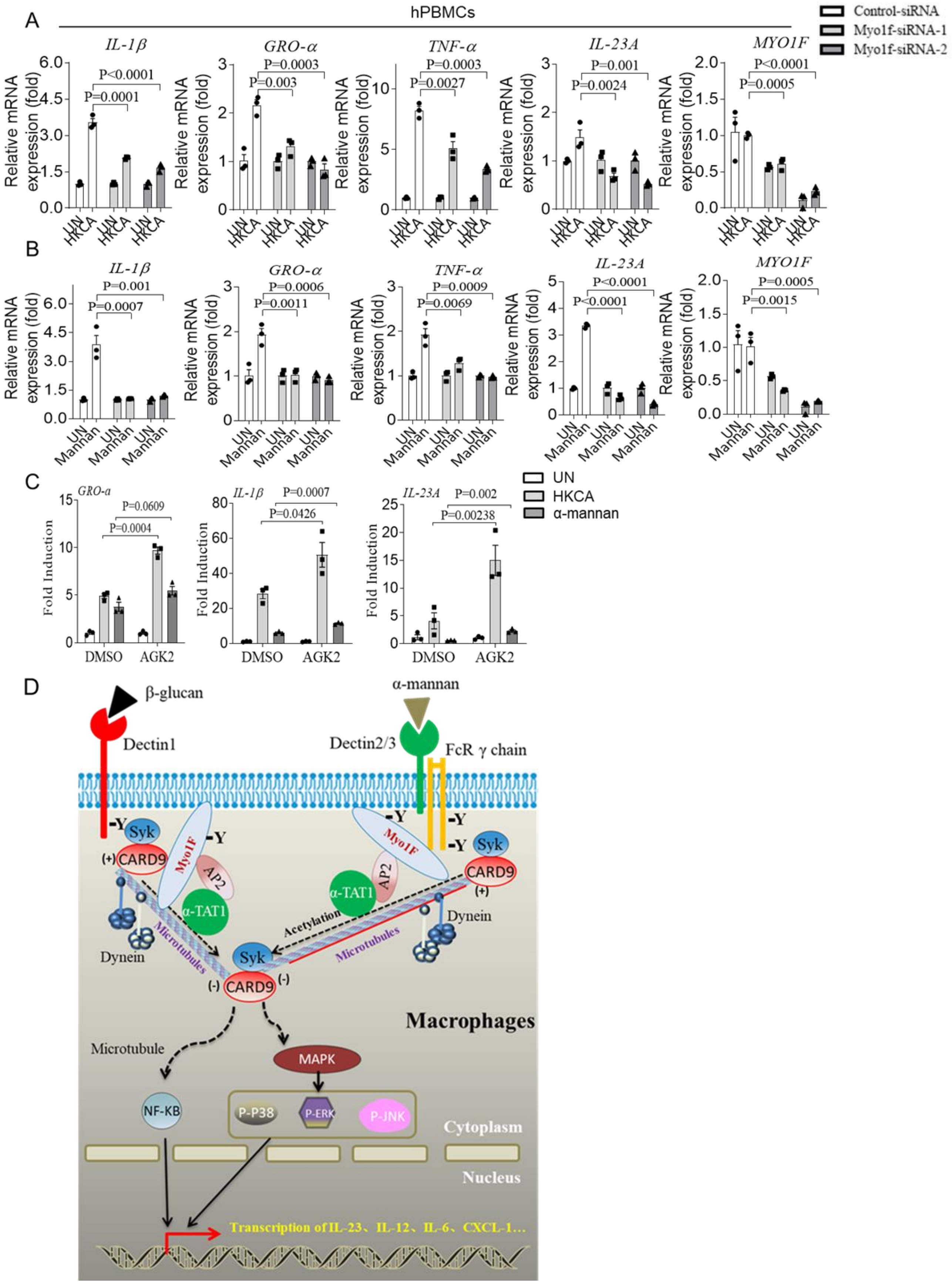
A
hPBMCs
Control-siRNA
Myo1f-siRNA-1
Myo1f-siRNA-2
IL-1β
GRO-α
TNF-α
IL-23A
MYO1F
Relative mRNA expression (fold)
P<0.0001
P=0.0001
P=0.0003
P=0.003
P=0.0003
P=0.0027
P=0.001
P=0.0024
P<0.0001
P=0.0005
UN
HKCA
B
IL-1β
GRO-α
TNF-α
IL-23A
MYO1F
P=0.001
P=0.0007
P=0.0006
P=0.0011
P=0.0009
P=0.0069
P<0.0001
P<0.0001
P=0.0005
P=0.0015
UN
Mannan
C
UN
HKCA
α-mannan
GRO-α
IL-1β
IL-23A
Fold Induction
P=0.0609
P=0.0004
P=0.0007
P=0.0426
P=0.002
P=0.00238
DMSO
AGK2
D
β-glucan
Dectin1
α-mannan
Dectin2/3
FcR γ chain
Syk
CARD9
Myo1F
AP2
α-TAT1
Dynein
Microtubules
Acetylation
Macrophages
Microtubule
MAPK
NF-KB
P-P38
P-ERK
P-JNK
Cytoplasm
Nucleus
Transcription of IL-23、IL-12、IL-6、CXCL-1…

Figure S8. MYO1F plays a critical role in the antifungal immunity in human PBMCs. (A-B) PBMCs from healthy donors were transfected with control siRNA or MYO1F siRNA, and 5 days after transfection, cells were treated with heat-killed *C. albicans* (HKCA, MOI=2) (**A**) or α-mannan (100 μg/ml) (**B**) for 3 hours, followed by real-time PCR analysis of indicated gene expression. **(C)** PBMCs from 3 healthy donors were pretreated with DMSO or AGK-2 (10 μM) for one hour, and stimulated with heat-killed *C. albicans* (HKCA, MOI=2) or α-mannan (100 μg/ml) for 3 hours, followed by real-time PCR analysis of indicated gene expression. (**D**) Overall model of functional role of MYO1F in antifungal signaling pathway was shown. MYO1F recruit AP2A1 and ATAT1 acetyltransferase complex for the α-Tubulin acylation after fungal stimulation, and acylated α-Tubulin is critical for the Syk and CARD9 translocation from membrane to the cytoplasm, which is an essential step for antifungal signaling activation. *: $P<0.05$; **: $P<0.01$; ***: $P<0.001$; ****: $P<0.0001$ based on two-tailed unpaired t-test (**C**), and one-way ANOVA for **A-B**. All error bars represent S.E.M of technical replicates. Data are representative of three independent experiments.

Legends for Dataset S1-S6

Dataset S1-S3. Mass spectrometry identification of TAGAP-interacting proteins. GST proteins (Dataset S1) or mouse GST-TAGAP proteins (Dataset S2-S3) were incubated with cell lysates of mouse BMDMs (Dataset S1-S2) or cell lysates of curdlan-treated BMDMs (100 μg/ml, 30 mins, Dataset S3) for 4 hours at 4 °C. After washing for four times with washing buffer, protein was eluted with lysis buffer containing 0.1% of SDS and analyzed by mass spectrometry analysis.

Dataset S4-S6. Mass spectrometry identification of MYO1F-interacting proteins. PCDH-EGFP-puro empty vector stable infected THP-1 cells (Dataset S4) and PCDH-EGFP-puro-Flag-hMYO1F stable infected THP-1 cells (Dataset S5-S6) were differentiated with PMA (25 ng/mL) for 3 days, and the cells were left untreated (Dataset S4-S5) or treated with HKCA for 30 mins (MOI=2, Dataset S6), followed by immunoprecipitation by anti-Flag antibody. After washing for four times with IP buffer, protein was eluted with IP buffer containing 0.1% of SDS and analyzed by mass spectrometry analysis.
